# Topology-generating interfacial pattern formation during liquid metal dealloying

**DOI:** 10.1038/ncomms9887

**Published:** 2015-11-19

**Authors:** Pierre-Antoine Geslin, Ian McCue, Bernard Gaskey, Jonah Erlebacher, Alain Karma

**Affiliations:** 1 Physics Department and Center for Interdisciplinary Research on Complex Systems, Northeastern University, Boston, Massachusetts 02115, USA; 2 Department of Materials Science and Engineering, Johns Hopkins University, 3400 North Charles Street, Baltimore, Maryland 21218, USA

## Abstract

Liquid metal dealloying has emerged as a novel technique to produce topologically complex nanoporous and nanocomposite structures with ultra-high interfacial area and other unique properties relevant for diverse material applications. This process is empirically known to require the selective dissolution of one element of a multicomponent solid alloy into a liquid metal to obtain desirable structures. However, how structures form is not known. Here we demonstrate, using mesoscale phase-field modelling and experiments, that nano/microstructural pattern formation during dealloying results from the interplay of (i) interfacial spinodal decomposition, forming compositional domain structures enriched in the immiscible element, and (ii) diffusion-coupled growth of the enriched solid phase and the liquid phase into the alloy. We highlight how those two basic mechanisms interact to yield a rich variety of topologically disconnected and connected structures. Moreover, we deduce scaling laws governing microstructural length scales and dealloying kinetics.

## Abstract (web summary)

Liquid metal dealloying produces topologically complex nanoporous structures, however little is known about how they form. Here, the authors use mesoscale phase-field modeling and experiments to show that structure formation is governed by both interfacial spinodal decomposition and diffusion-coupled growth.

## Graphical abstract

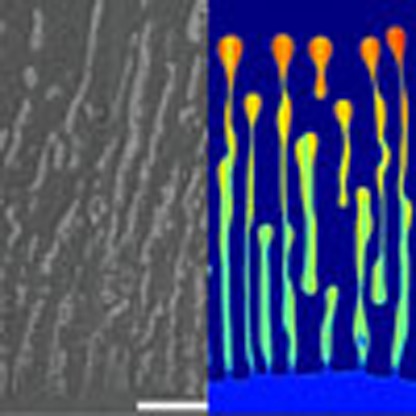

Dealloying is the selective dissolution of an element out of an alloy. Typically, it occurs as an undesired corrosion mechanism where the less noble component is dissolved away, leading to brittle crack propagation, stress corrosion cracking and other undesirable materials failure. One can, however, take advantage of this process by dissolving the less noble element of a binary alloy in an acid bath and produce a nanoporous structure of the more noble element^1^,^2^,^3^,^4^,^5^,^6^. This electrochemical dealloying technique has been used and studied extensively on some metallic alloys (Ag–Au, Cu–Au, Ni–Pt, Mn–Cu and Mn–Ni), to produce nanoporous structures with interesting properties of catalysis^7^,^8^, actuation^9^, sensing^10^, capacitors^11^ and radiation-damage tolerant materials^12^. However, the issue with electrochemical dealloying is that there are a limited number of alloys with a large-enough difference in reduction potential enabling porosity formation. In a recent breakthrough, Wada *et al.*^13^ generalized this process to a larger class of materials by using a liquid metal in lieu of the acid bath, leading to the formation of similar nano/microstructures. This liquid metal dealloying (LMD) technique relies on the choice of the liquid metal element (C), required to possess a high enthalpy of mixing with one of the elements of the precursor A–B alloy: the miscible element (B) is dissolved selectively in the liquid metal, while the immiscible element (A) simultaneously organizes and forms a porous structure. After solidification of the liquid metal, a nano/microcomposite of A-rich and B–C solid phases is formed, presenting excellent mechanical properties. In a second step, one of the phase can be etched away to obtain a nanoporous structure with a high specific area. Several new materials with outstanding properties have been fabricated with this new technique: nanoporous Si for battery anodes with extremely long cycle fatigue^14^, ultra-high surface area non-porous Nb for electrolytic capacitors^15^ and Cu–Ta nanocomposites with outstanding material properties^16^.

Pattern formation during dealloying remains poorly understood. Coarsening has been shown to contribute to the evolution of topologically connected structures away from the dealloying front^17^,^18^,^19^ but does not explain how structures form. In the context of electrochemical dealloying, it has been proposed that nanoporosity formation results from spinodal decomposition at the solid-electrolyte interface^1^ but testing this scenario has proven difficult. Previous atomistic studies using Kinetic and Metropolis Monte Carlo methods have been able to roughly reproduce topologically connected structures observed in electrochemical dealloying and the effect of electrochemical potential driving forces on these structures. However, these methods treat the details of the solid-electrolyte interface using simplistic assumptions for metal/anion interactions^1^,^2^,^20^,^21^,^22^,^23^. Moreover, they universally ignore the kinetics of the dissolved component in the dealloying medium, which we show here to play a crucial role in pattern formation during LMD. Electrochemical effects are extremely difficult to incorporate into phase-field models^24^,^25^, which has so far hindered a full quantitative description of electrochemical dealloying. In contrast to solid-electrolyte interfaces, the solid–liquid metal interface is better understood and readily captured by phase-field modelling. This allows us to address the outstanding question of what happens at the early stages of pattern formation and to explain the formation of a wide range of topologically connected and disconnected structures that we observe experimentally as a function of alloy composition during LMD.

In this study, we combine phase-field modelling and experiments to reveal the key mechanisms controlling the formation and evolution of topologically complex structures. Experiments are carried out by dealloying 
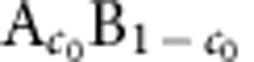
 alloys with an initial composition *c*_0_ in contact with pure C liquid, varying the immersion time into the melt to investigate different dealloyed depths. The elements are chosen to be Ta (A) and Ti (B) that are respectively immiscible and miscible in liquid Cu (C). Dealloying produces a bicontinuous nanocomposite structures made of A-rich and B–C phases that are analysed using scanning electron microscopy. Furthermore, we use a standard phase-field model of ternary alloys with parameters that approximate the Ta–Ti–Cu system. Simulations are carried out in both two and three dimensions (2D and 3D) to highlight topological differences dependent on dimensionality and to access longer time scales in 2D. The ability of the phase-field method to model complex interfacial patterns in solidification^26^,^27^,^28^,^29^ and other phase transformations^30^,^31^ makes it a method of choice to explore topologically complex dealloyed structures. Moreover, the simpler structure of the solid–liquid interfaces in metallic alloys, compared with alloy-electrolyte interfaces, makes the phase-field method readily able to investigate dealloying in the context of LMD. Using phase-field simulations and experiment, we show that interfacial spinodal decomposition destabilizes the dealloying front via the formation of compositional domains enriched in the immiscible element with an initial spacing of several nanometres. We further demonstrate that this mechanism is insufficient to explain the subsequent evolution of the interface pattern at the dealloying front to much larger scales ranging from hundreds of nanometres to tens of micrometres depending on the alloy composition, which is also not explained by coarsening occuring away from this front. We show that this interface pattern evolution is controlled by ‘diffusion-coupled growth' of solid domains enriched in the immiscible element and the liquid phase enriched in the miscible element. This pattern formation mechanism is remarkably analogous to the coupled growth of two-phase structures in solidification^32^ and other phase transformations^33^, but unexpected in LMD where alloys do not typically exhibit three-phase equilibria.

## Results

### Microstructure and topology selection

**Figure 1:**
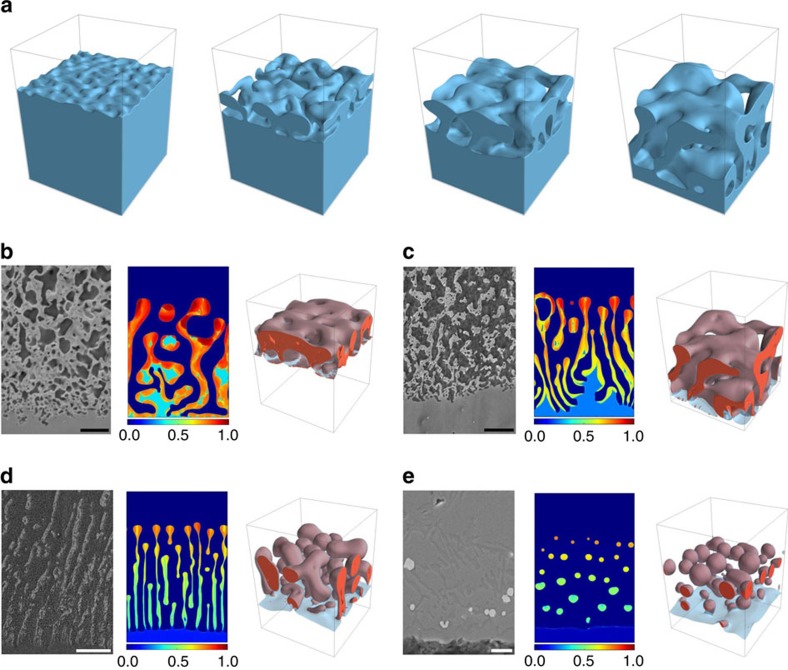
Microstructure and toplogy selection. (**a**) Phase-field simulation illustrating the formation of a topologically connected bicontinuous nanostructure during the dealloying of a A_*c*_0__B_1−*c*_0__ alloy in contact with a pure C liquid for *c*_0_=25%. Snapshots of the solid–liquid interface (*φ*=1/2 surface) are shown at dealloying times 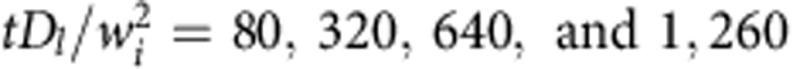. The simulation domain size is 96 × 96 × 128 nm^3^. Structures for *c*_0_=35% (**b**), *c*_0_=25% (**c**), *c*_0_=15% (**d**) and *c*_0_=5% (**e**) in experiments, and 2D and 3D simulations in domain sizes 256 × 384 nm^2^ and 96 × 96 × 128 nm^3^, respectively. 2D simulation results are shown by a colourmap of the concentration of the immiscible alloy element A. For 3D results, the solid–liquid interface is represented in transparent light blue, while the red surface represents an iso-concentration surface *c*_A_=1/2, delimiting the A-rich solid phase. The solid–liquid interface appears in light blue at the dealloying front and brown (due to colour addition of light blue and red) when the interface covers the A-rich phase. Scale bars, 1 μm (**b**), 3 μm (**c**), 10 μm (**d**) and 5 μm (**e**) (on experimental pictures, respectively).

Figure 1 illustrates the different topologies observed in simulations and experiments as a function of initial alloy composition *c*_0_. For high *c*_0_, a connected 3D structure of A-rich ligaments is formed. Figure 1a illustrates the evolution of the solid–liquid interface during the formation of this structure for *c*_0_=25%. This complex evolution yields a bicomposite of interpenetrating A-rich and B–C phases (also a nanoporous structure after the B–C phase is etched out). Interestingly, over the same composition range, ligaments of corresponding simulated 2D structures are disordered but not connected (Fig. 1b,c). For a lower intermediate range of *c*_0_, a 3D filamentary structure is formed consisting of disconnected A-rich filaments inside the B–C matrix, also manifested as a lamellar structure in 2D. Filaments or lamellae are aligned along an axis perpendicular to the dealloying front (Fig. 1d) and filaments can further break up into aligned blobs in 3D. For low *c*_0_, a globular structure is formed consisting of randomly dispersed 3D blobs (or 2D islands) of the A-rich phase in the B–C matrix (Fig. 1e). It is important to emphasize that topologically disconnected filamentary and blob structures do no have a direct analogue in electrochemical dealloying that yields connected structures even for very low composition, for example, as low as 2%Au in Ag–Au alloys^34^. Filaments and blobs during LMD result from interfacial pattern formation and not from topological changes induced by coarsening of an already formed connected structure. Simulations probe smaller dealloying depths than the experiments but are large enough to reproduce salient features of the observed topologies for different alloy compositions. Furthermore, they capture very early stages of interfacial pattern formation that are impossible to investigate experimentally with the currently available techniques.

### Initial destabilization

**Figure 2:**
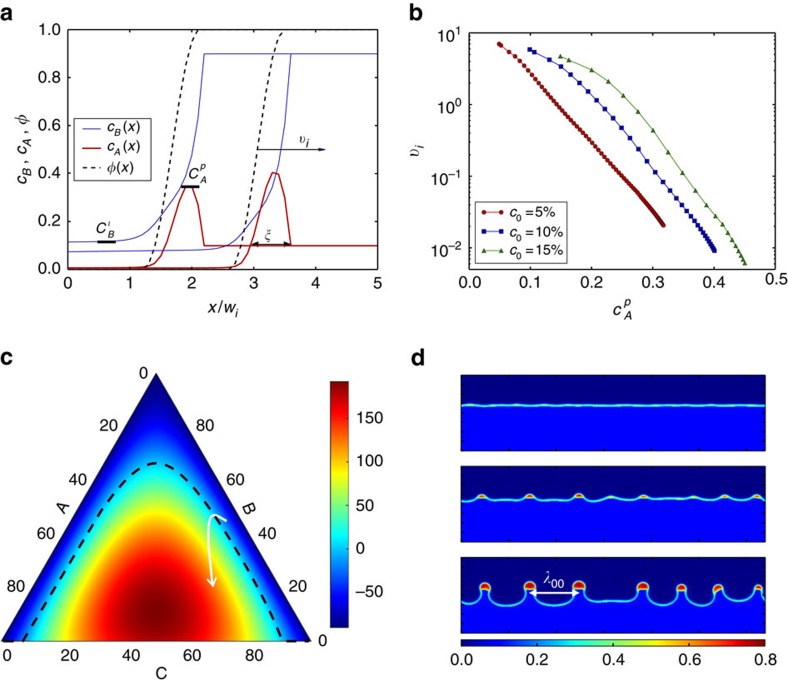
Interfacial spinodal decomposition. (**a**) Evolution of the concentration profiles of A (red) and B (blue), and phase-field *φ* (black dashed line) during melting of an A_*c*_0__B_1−*c*_0__ alloy with *c*_0_=10% in contact with a pure C liquid. The one-dimensional simulations model the advance of a planar interface in a semi-infinite system where *c*_B_=0 at *x*=−∞ and only the concentration profiles close to the solid–liquid interface are shown for clarity. (**b**) Semi-log plots of the solid–liquid interface velocity *v*_*i*_ versus the peak interfacial value 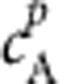 of the concentration *c*_A_ of the immiscible alloy element for different *c*_0_. (**c**) Ternary plot of the driving force *f*_*s*_ for spinodal decomposition showing the boundary (*f*_*s*_=0 with a black dashed line) between thermodynamically unstable (*f*_*s*_>0) and stable (*f*_*s*_<0) regions, and superimposed trajectory (white line) of the interfacial concentrations (taken at *φ*=0.5). (**d**) Snapshots of a 2D simulation (64 × 16 nm^2^) for *c*_0_=10% showing the formation of A-rich compositional domains and the resulting corrugation of the solid–liquid interface.

Figure 2 highlights the mechanism controlling the initial destabilization of a planar dealloying front. Figure 2a first shows the evolution of the concentration profiles within the solid–liquid interface region for a planar dealloying front in a one-dimensional simulation geometry. The immiscible alloy element A that is rejected by the solid on melting is constrained to remain inside the solid–liquid interfacial layer, leading to a build up of that element accompanied by the formation of a concentration peak denoted by 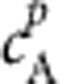 and a decrease of interface velocity *v*_*i*_. In contrast, the miscible element is able to diffuse away in the liquid, decreasing in concentration from a value 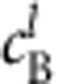 on the liquid side of the interface to a value 
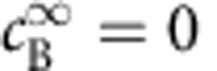
 corresponding to pure C far on the liquid side of the interface (*x*→−∞ in Fig. 2a). The alloy solidification or melting rate is generally controlled by diffusive atomic transport away from the interface and attachment/detachment kinetics of atoms at the interface, with the former and latter being dominant for low and high solidification/melting rate, respectively^32^,^33^. In particular, a high solidification rate of the order of m s^−1^ is known to lead to solute trapping in metallic alloys^35^,^36^ due to a strong departure from chemical equilibrium at the interface. In contrast, in dealloying simulations and experiments, the interface velocity (varying from mm s^−1^ to μm s^−1^) is small enough for the interfacial concentration profiles to relax quickly to quasi-local thermodynamic equilibrium so that 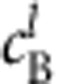 is determined predominantly by 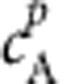 and *v*_*i*_ is controlled by diffuse transport of the miscible B element in the liquid away from the interface. A detailed analysis of chemical equilibrium at the interface shows that *c*_B_^*l*^ is a strongly decreasing function of 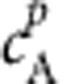 (see Supplementary Note 1 and Supplementary Fig. 1), with the precise relationship depending generally on the ternary phase diagram and the coefficients *σ*_*i*_ of gradient squared terms in the free energy. Furthermore, mass conservation implies that the interface velocity is proportional to the magnitude of the concentration gradient of B on the liquid side of the interface, which decreases when 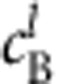 decreases. As a result, similar to 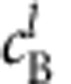, *v*_*i*_ is a strongly decreasing function of 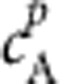 (Fig. 2b). This decrease is approximately fitted by an exponential function 
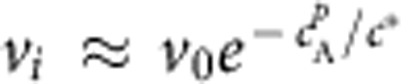
 for the present simulation parameters with 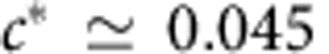 weakly dependent on *c*_0_ and *v*_0_ increasing with *c*_0_.

For a one-dimensional planar interface, this exponential decay of the velocity causes dealloying to stall after dissolution of a few solid atomic layers. In contrast, in 2D or 3D the buildup of the immiscible alloy element inside the interfacial layer can cause phase separation into A-rich and A-poor compositional domains to occur laterally along the interface when the interfacial compositions exceeds the threshold for spinodal decomposition. This threshold can be estimated by a linear stability analysis of small variations around uniform concentrations^37^,^38^, which predicts that the driving force for spinodal decomposition in a ternary system is given by 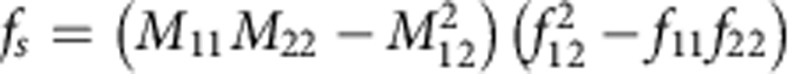 (where *M*_*ij*_ are components of the mobility matrix and 
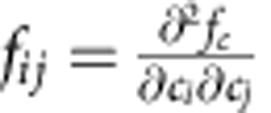
, *f*_*c*_ being the chemical free energy defined in Supplementary Note 2). We plot this driving force as a function of concentrations in Fig. 2c using the standard Gibbs triangle representation for ternary systems. The boundary between unstable (*f*_*s*_>0) and stable (*f*_*s*_<0) regions inside the triangle is shown by a black dashed line (*f*_*s*_=0). Superimposed on this plot is the trajectory (white line) corresponding to the time variation of interfacial concentrations (*c*_A_, *c*_B_, *c*_C_=1−*c*_A_−*c*_B_ evaluated at *φ*=1/2) during the planar front simulation of Fig. 2a for *c*_0_=10%. This trajectory crosses the spinodal boundary, thereby leading to the formation of A-rich compositional domains along the interface with an initial spacing *λ*_00_ as illustrated in the 2D simulation of Fig. 2d. As the interface velocity strongly decreases with 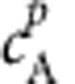 (Fig. 2b), dissolution stalls along A-rich domains but continues in between those domains, causing the solid–liquid interface to become morphologically unstable with a wavelength ∼*λ*_00_. This scale is inversely proportional to *c*_0_ for *c*_0_≤10% and saturates to ∼8–10 nm for *c*_0_≥15% in both 2D and 3D simulations.

### Diffusion-coupled growth

**Figure 3:**
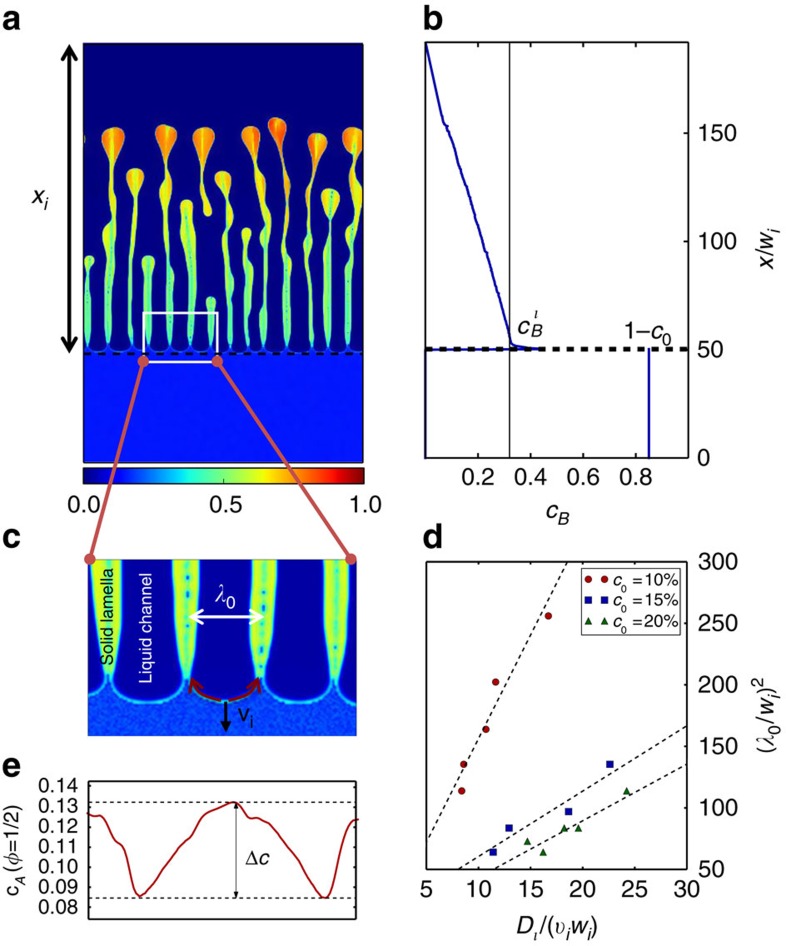
Diffusion-coupled growth. (**a**) Example of diffusion-coupled growth of a lamellar structure in a 2D phase-field simulation for *c*_0_=15%. (**b**) Plot of the horizontally averaged concentration of the miscible element B showing that the interface velocity *v*_*i*_ is controlled by the diffusion of B in the liquid phase. (**c**) Magnified view of the lamellar structure with arrows indicating the lateral diffusion of A along the solid–liquid interface that mediates the coupled growth of the A-rich solid and B–C liquid phase. (**d**) Concentration profile along the solid–liquid interface (*φ*=0.5) for the segment of interface in between the two arrows in **c**. (**e**) Plots validating the scaling law 
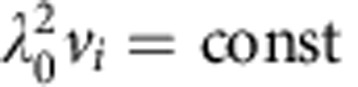
 where the constant depends on alloy composition and the dashed straight lines are linear fits; *λ*_0_ increases with dealloying depth *x*_*i*_ due to a decrease of the magnitude of the concentration gradient of B in liquid and hence *v*_*i*_.

Although interfacial phase separation explains the initial stage of pattern formation, it does not predict the subsequent pattern evolution on much larger scales than the initial domain spacing. We propose an analysis to understand this evolution for the lamellar/filamentary 2D/3D structures of Fig. 1d, which form over an intermediate range of *c*_0_ and grow without recurrence of interfacial spinodal decomposition. The growth mechanism of the lamellar structure is highlighted in Fig. 3 using 2D simulations that access larger dealloying depths. Figure 3a shows that the interface pattern at the dealloying front is strikingly similar to the classical lamellar structures formed during eutectic transformation of a liquid into two solid phases^32^,^33^,^39^,^40^. Eutectic growth, and more broadly diffusion-coupled growth of two compositionally distinct phases into another phase of uniform composition (for example, solid/liquid into liquid during crystallization of monotectic alloys^41^ or solid/solid into solid during eutectoid phase transformations^33^), is controlled by lateral diffusion of solute species ahead of the two-phase front. In the classic example of eutectic solidification of a binary AB alloy, the A/B element rejected by the B-rich/A-rich solid phase diffuses through the liquid to the adjacent A-rich/B-rich solid phase where the element is incorporated, enabling the coupled (that is, cooperative) growth of both phases. A similar mechanism is revealed by our dealloying simulations where the A-rich solid lamellae reject B atoms that diffuse to the adjacent B–C liquid channels and, *vice versa*, those channels reject A atoms that diffuse to the A-rich solid lamellae as indicated by the arrows in Fig. 3c so that both the A-rich solid phase and B–C liquid channels grow cooperatively into the A–B solid alloy. Although two-phase eutectic/monotectic/eutectoid coupled growth generally involves diffusion in bulk phases, lateral diffusion in the LMD analogue is confined to the interfacial concentration boundary layer of thickness *ξ* (illustrated in Fig. 2a), where *ξ* is a nanometric length of the order of the solid–liquid interface thickness. For coupled growth to occur at velocity *v*_*i*_, the flux of A elements into the interfacial layer produced by the growth of the B–C liquid phase into the alloy 
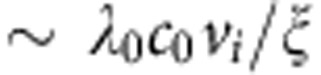
 must equal the flux of A into the A-rich solid mediated by lateral diffusion along this layer 
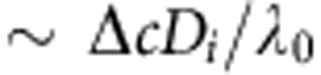
, where Δ*c* is the difference of concentration of A between the tip of a liquid channel and the edges of nearby A-rich lamellae (see Fig. 3e and Supplementary Fig. 2) and *D*_*i*_ is the interfacial diffusivity at *φ*=1/2 (*D*_*i*_=*D*_*l*_/2 for the choice of mobility in the phase-field model). Equating those two fluxes yields the scaling law 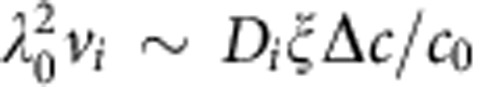. This law predicts that the lamellar spacing 
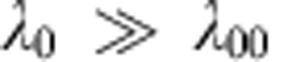
 increases with dealloying depth, as *v*_*i*_ decreases due to the progressive decrease of the concentration gradient of the miscible element B in the liquid, and is well obeyed in our simulations as shown in Fig. 3d.

**Figure 4:**
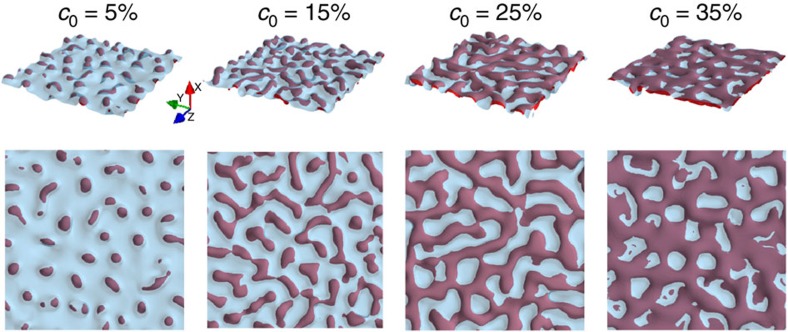
Topology of compositional domains. Snapshots (96 × 96 nm^3^) of 3D phase-field simulations highlighting the solid–liquid interface and compositional domains at the early stage of dealloying with the same colour coding as in Fig. 1. Domains become progressively more connected within the interfacial layer with increasing initial alloy composition *c*_0_ and seed, the growth of topologically distinct globular, filamentary, and connected structures (Fig. 1b–e).

The two mechanisms of interfacial spinodal decomposition and diffusion-coupled growth highlighted thus far provide the conceptual framework to understand the different topologies selected as a function of alloy composition and dimensionality. In a low composition range (for example, *c*_0_=5% and Fig. 1e), dimensionality does not play a crucial role. In this range, topologically disconnected A-rich domains first form by interfacial spinodal decomposition but the concentration of A is not high enough to maintain diffusion-coupled growth of either 2D lamellar or 3D filamentary structures. As a result, A-rich domains stop growing cooperatively with the liquid phase and detach from the dealloying front, after which new domains are formed again by interfacial spinodal decomposition. Cyclical formation/domain detachment then yields a disordered structure of dispersed 3D blobs or 2D islands (see Supplementary Movies 1 and 5). For an intermediate range of *c*_0_, initially formed A-rich domains are still largely disconnected, as illustrated in Fig. 4, which compares initial domain structures for different initial compositions. However, in this range the concentration of A becomes sufficient to maintain diffusion-coupled growth that allows dealloying to progress without recurrence of spinodal decomposition (see Supplementary Movie 2). As for eutectic coupled growth of two phases of strongly unequal volume fractions, dimensionality controls the morphology, which is lamellar and filamentary (rod-like) in 2D and 3D, respectively. Dimensionality also influences the subsequent evolution of the morphology, leading to the breakup of filaments by the surface tension driven Rayleigh–Plateau instability of liquid jets^42^ (see Supplementary Movie 6), also known to cause the breakup of rod-like two-phase eutectic structures^43^ or cylindrical liquid inclusions^44^ in metallic alloys. For larger *c*_0_, dimensionality plays a crucial role in topology selection. In 2D simulations, A-rich domains do not connect initially and spinodal decomposition recurs during growth in regions of the interface in between already formed A-rich domains. Newly formed A-rich domains can deflect and split the trajectories of liquid channels, yielding a disordered lamellar structure of curved A-rich lamellae (see Supplementary Movies 3 and 4). In contrast in 3D simulations, A-rich domains are able to connect within the two-dimensional interfacial layer (Fig. 4). Connected domains then grow by a topologically complex form of diffusion-coupled growth (see Supplementary Movies 7 and 8).

### Bridging scales between simulations and experiments

To relate simulations and experiments more quantitatively, we derive a relation to predict the increase of the interface pattern scale *λ*_0_ with the dealloying depth *x*_*i*_. Using a simple analytical model of diffusion-coupled growth detailed in Supplementary Note 3, which assumes a flat solid–liquid interface in between A-rich compositional domains, we obtain the relation


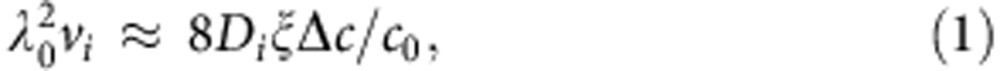


which includes the numerical prefactor to the scaling law derived earlier from flux balance. The dealloying front velocity *v*_*i*_ is controlled by the diffusion of B (Ti) in C (Cu). This is demonstrated in the discussion provided in Supplementary Note 4 and by Supplementary Fig. 3, showing that the activation energy of the process is close to the one of Ti diffusion in Cu.

Moreover, Supplementary Figs 4 and 5 (experimental and simulated, respectively) show that the concentration of B on the liquid side of the interface 
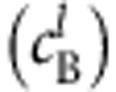
 can be considered constant during the dealloying process. This is a consequence of the aforementioned fact that the interface velocity is small enough for the interfacial concentration profiles to relax quickly to quasi-local thermodynamic equilibrium. More informations about this interface equilibrium are provided in Supplementary Note 1.

Finally, the concentration profiles provided in Supplementary Figs 4 and 5 show that *c*_B_ can be considered to vary linearly between 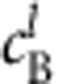 at the dealloying front and zero at the edge of the dealloyed structure. Mass conservation at the interface then implies that 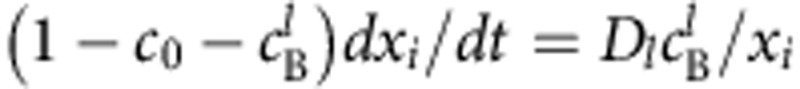, yielding


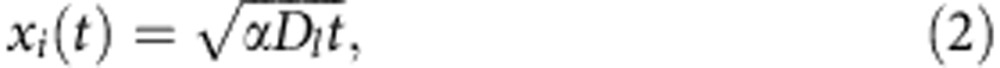


where 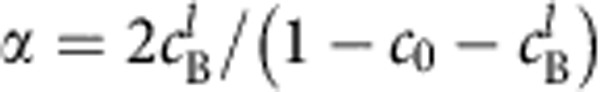 (see Supplementary Notes 5 and 6 for details). We note that relaxing the assumption that *c*_B_=0 at the edge of the dealloyed structure by assuming diffusion in an infinite liquid bath yields the same prediction with a different value of *α*. Combining equation (1) and equation (2), we obtain the desired relation between the interface pattern scale *λ*_0_ and the dealloyed depth *x*_*i*_


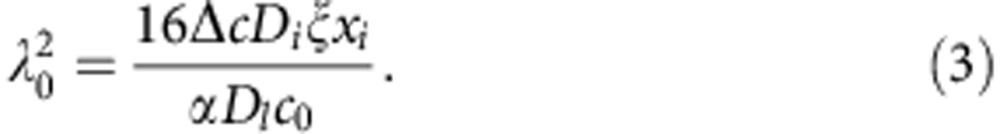


**Figure 5:**
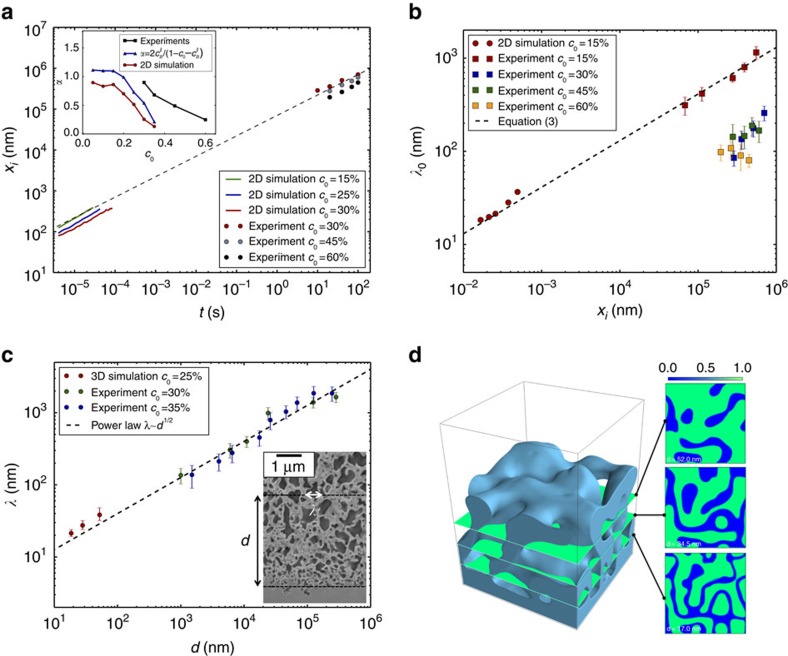
Scaling laws for dealloying kinetics and pattern length scales. (**a**) Log–log plot of dealloying depth *x*_*i*_ versus time from simulations (lines) and experiments (dots) for different alloy composition. The dashed black line represents a power law with the characteristic 1/2 exponent of diffusion-limited kinetics. (**b**) Interface pattern scale at the dealloying front *λ*_0_ versus dealloyed depth *x*_*i*_ showing agreement of simulations and experiments with theory (equation (3) is represented with a dashed line) over four decades of length scales for diffusion-coupled growth of lamellar/filamentary disconnected structures (*c*_0_=15%). Topologically complex connected structures for higher *c*_0_ exhibit an increase of *λ*_0_ with dealloyed depth, but with *λ*_0_ smaller than predicted by equation (3) that no longer holds. (**c**) Log–log plot of *λ* versus *d* where *λ*≥*λ*_0_ denotes the structure scale at a distance *d* from the dealloying front increased by coarsening for connected structures. Simulations and experiments follow the predicted coarsening behaviour with a 1/2 exponent. (**d**) Simulation snapshot and cross-sections showing the coarsening of the connected structure obtained in 3D simulation for *c*_0_=25%. Error bars on the experimental data points represent the s.d. obtained from 20 measurements.

The results reported in Fig. 5 show that those theoretical predictions agree quantitatively well with both simulation and experimental results. Because of computational limitations, simulations cannot be performed on the same scale than the experiments but the scaling laws of equations (2) and (3) allows us to bridge computational and experimental length and time scales over several orders of magnitude.

Figure 5a plots the dealloying depth as a function of time for both 2D simulations (where we take *D*_*l*_=7 × 10^−9^ m^2^ s^−1^ (ref. 45)) and experiments for different *c*_0_ showing that 
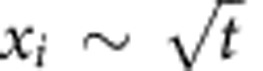
. Values of *α* extracted from experimental and numerical results are shown to be in reasonable agreement with the theoretical prediction of equation (2) with *α* estimated by extracting 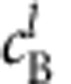 from simulations. Both simulations and experiments exhibit a decrease of 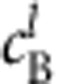 and hence *α* with *c*_0_ shown in the inset of Fig. 5a. Quantitative differences of *α*-values between theory, computations and experiments are relatively small given uncertainties in material parameters entering the model. The prediction of equation (3) is plotted with a dashed line in Fig. 5b where *D*_*i*_=*D*_*l*_/2, Δ*c* and *ξ* have been estimated from the analysis of the interfacial concentration profile (see Supplementary Note 3). For the alloy composition *c*_0_=15% forming lamellar/filamentary diffusion-coupled growth structures, theory predicts remarkably well the increase of A-rich domain spacing *λ*_0_ with dealloying depth over four decades of length scales. For such structures, diffusion-controlled coarsening of the dealloyed structure is slow and contributes primarily to the breakup of filaments.

For topologically connected structures forming at large alloy composition (*c*_0_≥30%), the experimental results displayed in Fig. 5b show that *λ*_0_ also increases with the dealloyed depth but equation (3) does not predict the length-scale evolution of these structures. This suggests that a more complex growth mechanism involving both diffusion-coupled growth and recurrent interfacial spinodal decomposition might contribute to smaller values of *λ*_0_ for topologically connected structures. As disconnected filamentary structures grow without recurrence of interfacial spinodal decomposition, they can increase their spacing continuously with increasing dealloyed depth as described by equation (3). However, recurrence of interfacial spinodal decomposition, which is promoted by a higher *c*_0_, would limit this spacing by creating new solid ligaments. This recurrence is observed in 2D simulations (for example, *c*_0_=25%) and would be expected in larger 3D simulations. Exploring this mechanism and developing a theory to predict *λ*_0_ for connected structures is an important task for future studies.

### Coarsening of topologically connected structures

In addition to influencing *λ*_0_, connectedness also enables much faster coarsening away from the dealloying front (visible in the inset of Fig. 5c). Hence, for bicontinuous structures, coarsening becomes the dominant mechanism controlling the final length scale in 3D. This coarsening has been previously characterized by a power law 
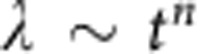
 with *n*≈1/4 (refs 17, 18, 19). Because of diffusion-limited kinetics, the dealloying depth evolves with a 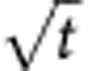 behavior and the length scale is expected to follow a 
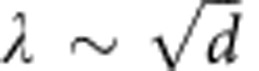
 behaviour, where *d* is the distance from the dealloying front. Figure 5c shows that both 3D simulations, with *λ* extracted from sections at different distances from the dealloying front (Fig. 5d), and experimental observations are in good agreement with this prediction.

In summary, our results demonstrate that two ubiquitous mechanisms of pattern formation in materials, spinodal decomposition and diffusion-coupled growth combine in the unique setting of LMD to generate topologically complex and varied structures on a wide range of scales. The first mechanism leads to compositional domain formation on a nanometre scale, whereas the second enables the simultaneous growth of compositionally distinct solid and liquid phases to larger nano/microscales. Topology is largely controlled by the initial alloy composition that dictates domain connectedness. As solidification of multicomponent alloys is known to produce extremely varied spatial arrangements of three or more simultaneously growing phases by diffusion-coupled growth^46^,^47^, the present finding that a similar mechanism controls interfacial pattern formation during LMD opens new avenues for creating even more complex and varied structures by this technique.

## Methods

### Experiments

For the experimental part of this study, Ti–Ta alloys of different compositions were immersed in a liquid bath of Cu under isothermal conditions at the temperature 1,513 K. This system was chosen for the following properties: Ti and Ta form a solid solution with a body-centred cubic symmetry across the entire composition range explored in this study; Ta is immiscible with Cu and molten Cu has a high Ti solubility. During dealloying, molten Cu selectively dissolves Ti out of the parent Ti–Ta alloy, while Ta diffuses along the metal/liquid interface and form different morphologies depending on the initial composition of the alloy. We use two different experimental setup: immersion experiments where the sample is dipped into the liquid Cu for a controlled period of time (see Supplementary Fig. 6a) and static experiments where the sample remains at the bottom of the crucible during the duration of the experiment (see Supplementary Fig. 6b). In both cases, on cooling the system we obtain a composite made of Ta-rich and Cu–Ti phases, whose morphology and structure are characterized using scanning electron microscopy. More details concerning the dealloying process and characterization are provided in the Supplementary Note 7.

### Phase-field model

Dealloying is modelled using the phase-field method that is well developed for ternary alloys (for example, see refs 48, 49). The model couples a phase field *φ* that distinguishes between the solid and liquid phases to the concentration fields representing the atomic fractions of the three elements with *c*_A_+*c*_B_+*c*_C_=1. The evolution equations for those fields are derived from a free-energy functional 
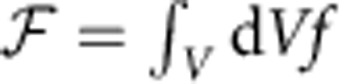
 (where *f* is the free-energy density), using standard variational forms for non-conserved and conserved dynamics


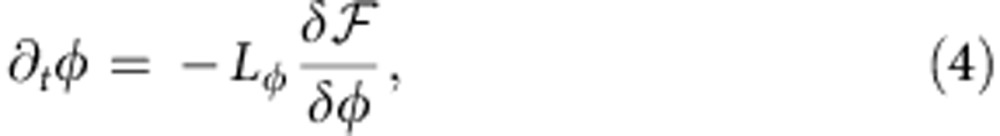



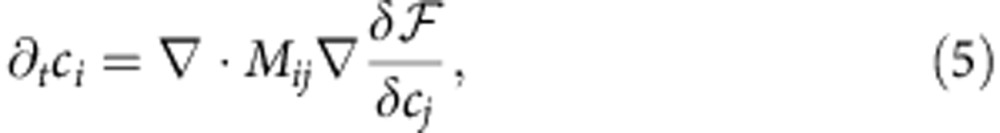


respectively. The free-energy density is expressed as the sum of (i) a double-obstacle potential function of *φ* and a gradient squared term 
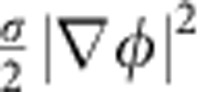
, which constrains the atomically rough solid–liquid interface to have a finite thickness *w*_*i*_, (ii) a bulk free-energy density contribution *f*_*c*_(*φ*, {*c*_*i*_}) chosen to reproduce approximately the thermodynamic properties of a mixture of Ta (A), Ti (B) and Cu (C), with a high enthalpy of mixing between the immiscible solid element A and liquid element C (phase diagrams of the binary systems are presented in Supplementary Fig. 7) and gradient squared terms in the concentration fields 
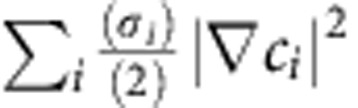
. The double-obstacle barrier height *σ* and *σ*_*i*_ are chosen to match typical excess free energies of the solid–liquid interface and compositional domain boundaries in metallic systems. The mobility matrix *M*_*ij*_=*M*_*l*_(1−*φ*)*c*_*i*_(*δ*_*ij*_−*c*_*j*_), where *δ*_*ij*_ is the Kronecker delta, yields vanishing diffusivity in the solid and Fickian diffusion in the liquid, with *M*_*l*_ chosen to match the experimental estimate of liquid-state diffusivity, *D*_*l*_. Finally, we choose 
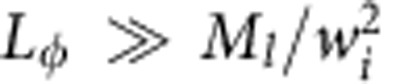
 consistent with the fact that the attachment/detachment kinetics of atoms at the solid–liquid interface in metallic alloys is fast compared with the liquid-state diffusion^33^. We have checked that results are independent of *L*_*φ*_ in this range. The parameter values chosen for the simulations are summarized in Supplementary Table 1 and additional informations on the phase-field model are provided in the Supplementary Note 2.

## Additional information

**How to cite this article:** Geslin, P.-A. *et al.* Topology-generating interfacial pattern formation during liquid metal dealloying. *Nat. Commun.* 6:8887 doi: 10.1038/ncomms9887 (2015).

## Supplementary Material

Supplementary Information Supplementary Figures 1-7, Supplementary Table 1, Supplementary Notes 1-7 and Supplementary References

Supplementary Movie 1 This movie presents a 2D simulation (256 × 384 nm2) of the dealloying of a AB alloy with composition c0 = 5% in A in contact with pure C liquid, leading to the formation of non-connected islands.

Supplementary Movie 2 This movie presents a 2D simulation (256 × 384 nm2) of the dealloying of a AB alloy with composition c0 = 15% in A in contact with pure C liquid, leading to the formation of elongated morphologies through the diffusion-coupled growth mechanism.

Supplementary Movie 3 This movie presents a 2D simulation (256 × 384 nm2) of the dealloying of a AB alloy with composition c0 = 25% in A in contact with pure C liquid, leading to the formation of a disordered structure.

Supplementary Movie 4 This movie presents a 2D simulation (256 × 384 nm2) of the dealloying of a AB alloy with composition c0 = 35% in A in contact with pure C liquid, leading to the formation of a disordered structure.

Supplementary Movie 5 This movie presents a 3D simulation (96 × 96 × 128 nm3) of the dealloying of a AB alloy with composition c0 = 5% in A in contact with pure C liquid, leading to the formation of non-connected blobs.

Supplementary Movie 6 This movie presents a 3D simulation (96 × 96 × 128 nm3) of the dealloying of a AB alloy with composition c0 = 15% in A in contact with pure C liquid, leading to the formation of elongated non-connected morphologies

Supplementary Movie 7 This movie presents a 3D simulation (96 × 96 × 128 nm3) of the dealloying of a AB alloy with composition c0 = 25% in A in contact with pure C liquid, leading to the formation of a nanoporous connected structure.

Supplementary Movie 8 This movie presents a 3D simulation (96 × 96 × 128 nm3) of the dealloying of a AB alloy with composition c0 = 35% in A in contact with pure C liquid, leading to the formation of a nanoporous connected structure.
