# Supplementary material for: Topology-generating interfacial pattern formation during liquid metal dealloying
Source: Nat Commun. 2015 Nov 19;6:8887. doi: 10.1038/ncomms9887 (PMC4673498; doi:10.1038/ncomms9887)
Supplement: Supplementary Information — Supplementary Figures 1-7, Supplementary Table 1, Supplementary Notes 1-7 and Supplementary References [file ncomms9887-s1.pdf]

Supplementary Figures

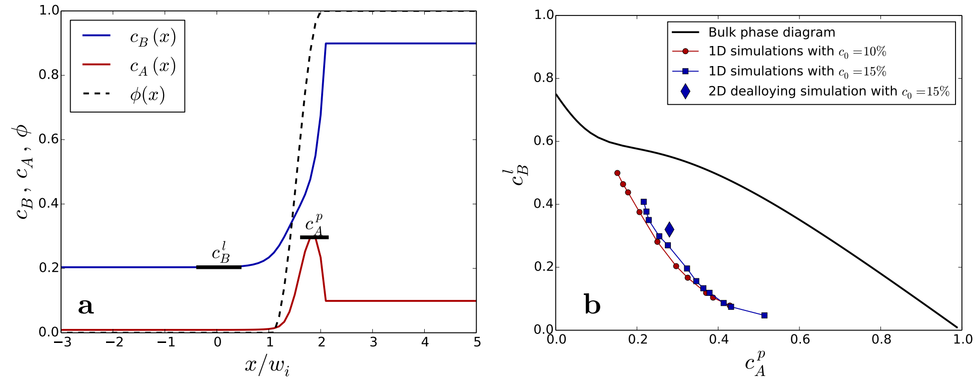

Supplementary Figure 1: Interface equilibrium. a. Concentration profile in the case of interface equilibrium. **b.** Relation between the interfacial peak concentration of A (Ta) noted c_A^p and the concentration of B (Ti) in the liquid, noted c_B^l. See Supplementary Note 1 for details.

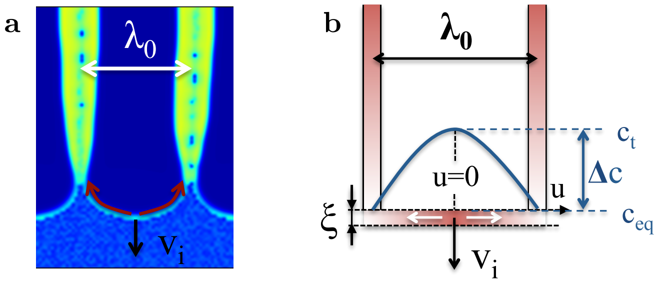

Supplementary Figure 2: Modeling of the diffusion-coupled growth. a. Coupled growth observed during the dealloying of an alloy with composition $c_0 = 15\%$. **b.** Simple model of 1D diffusion for the coupled growth assuming a planar dealloying front.

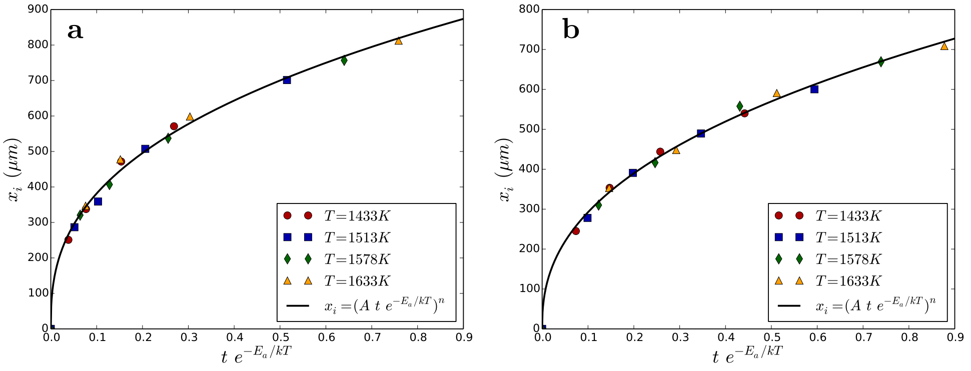

Supplementary Figure 3: Dealloying kinetics and activation energy. Dealloying depth function of the reduced time $te^{-Ea/kT}$ with $E_a = 0.69$ eV for alloy compositions $c_0 = 30\%$ (**a**) and $c_0 = 45\%$ (**b**), showing that the activation energy of the dealloying process is very close to Ti diffusion in Cu liquid: $E_{Ti\rightarrow Cu} = 0.715$ eV [1].

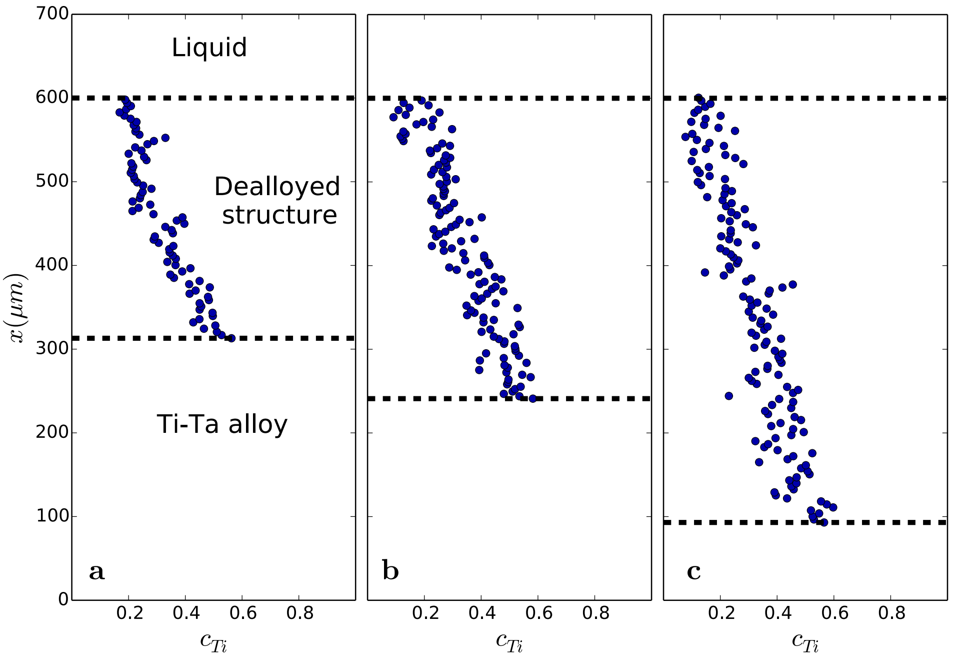

Supplementary Figure 4: Experimental concentration profiles. Profiles of Ti in the Ti/Cu phase of the dealloyed structure for three different dealloying times: $t = 10$ sec (**a**), $t = 20$ sec (**b**) and $t = 40$ sec (**c**) . The initial Ti-Ta alloy has a composition $c_0 = 30\%$. The origin of the x axis is taken at an arbitrary position in the solid. The lower dashed line represents the dealloying front (border between the Ti-Ta alloy and the dealloyed structure) while the upper dashed line represents the edge of the dealloyed structure (border between the dealloyed structure and the liquid).

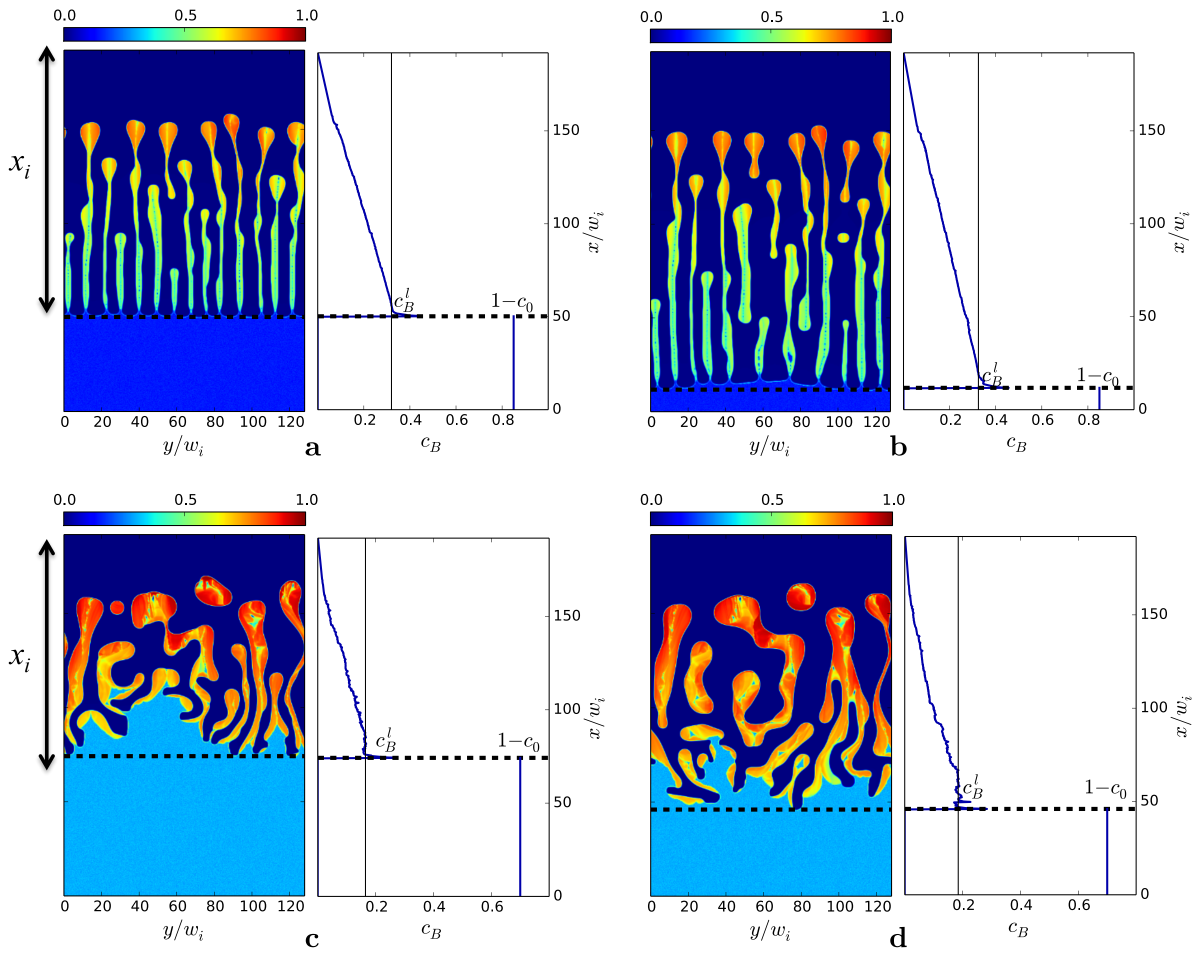

Supplementary Figure 5: Concentration profiles obtained numerically. Simulation snapshots (representing the concentration field c_A) and concentration profiles of B in the B-C liquid phase for an initial composition:
(**a-b**) $c_0 = 15\%$ at dealloying times $t = 3 \cdot 10^3 w_i^2/D_l$ (**a**) and $t = 4.5 \cdot 10^3 w_i^2/D_l$ (**b**)
(**c-d**) $c_0 = 30\%$ at dealloying times $t = 6 \cdot 10^3 w_i^2/D_l$ (**c**) and $t = 9 \cdot 10^3 w_i^2/D_l$ (**d**).

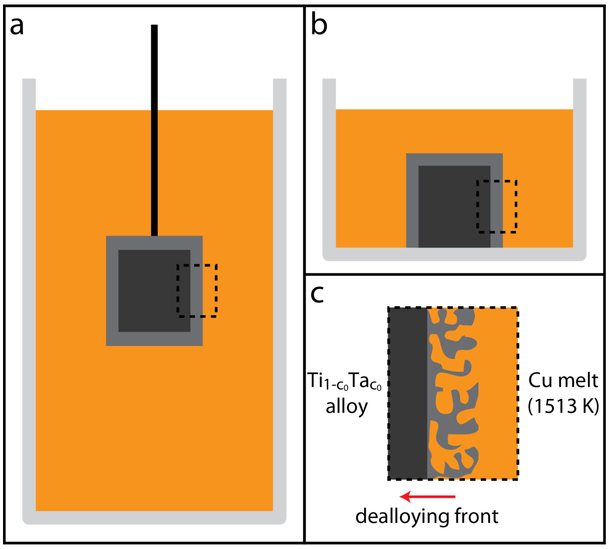

Supplementary Figure 6: Liquid metal dealloying experiments. a. Immersion experiment where the sample is dipped into a bath of molten Cu and then removed after a fixed time. **b.** Static experiment where the sample remains at the bottom of the crucible during the dealloying process and during the solidification of the Cu bath. **c.** Zoom-in of the dealloyed regions, illustrating the dealloying direction, edge of the sample, and the initial Ti-Ta alloy.

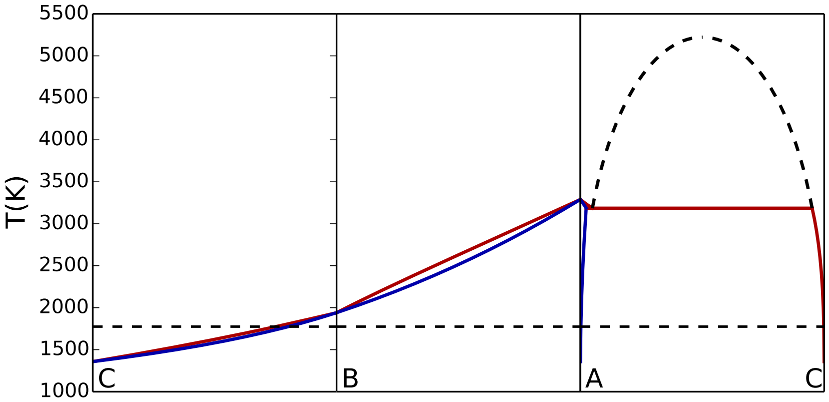

Supplementary Figure 7: Phase diagrams. The phase diagrams of the binary systems (C,B) (B,A) and (A,C) are computed from the free energy of the phase-field model. The solidus (liquidus) lines are represented in blue (red). Phase separation in the liquid for the phase diagram (A,C) is shown with a dashed line.

T_{sim}	1775 K
V_a	0.01 nm^3
σ_ϕ	3.18 eV nm^{-1}
λ_ϕ	1.59 eV nm^{-3}
L_A, L_B, L_C	$17.6, 11.8, 11.5$ eV nm^{-3}
T_A, T_B, T_C	$3290, 1941, 1358$ K
σ_A, σ_B, σ_C	$9.0, 9.0, 9.0$ eV nm^{-1}
Ω	90 eV nm^{-3}
D_l	$7 \cdot 10^9$ nm^2 sec^{-1}
L_ϕ	$1.14 \cdot 10^9$ $\mathrm{nm}^3\mathrm{eV}^{-1}\mathrm{s}^{-1}$

Supplementary Table 1: Parameters. List of the parameter values for the phase-field model.

Supplementary Note 1: Equilibrium condition at the dealloying front

Numerical results show that the concentration of the dealloyed element B (Ti) on the liquid side of the dealloying front is controlled by an equilibrium condition. During the dealloying process, a layer of A (Ta) remains at the solid side of the interface (see for example Fig. 3.c of the main article), and governs the concentration of B (Ti) at the liquid side of the dealloying front.

To investigate this equilibrium relation, we perform 1D simulations starting with a A-B alloy with composition $c_0 = 10\%$ in contact with a liquid of pure C. A "no flux" boundary condition is imposed on the liquid side of the system and different simulations are performed with various system sizes. The solid/liquid interface moves until an equilibrium is reached between the peak of A accumulated at the solid/liquid interface and the concentration of B (Ti) in the liquid. For large system sizes, the reservoir of liquid is bigger and the interface moves a greater distance before reaching equilibrium, building up a higher peak of Ta. Thus, different system sizes corresponds to different equilibrium relations the interface.

Supplementary Fig. 1.a shows the concentration profile of composition B and C after reaching an equilibrium state. The height of the peak of A noted c_A^p and the concentration of B (Ti) at the liquid side of the interface, noted c_B^l are then reported on Supplementary Fig. 1.b.

This relation can be compared to prediction extracted from the bulk ternary phase diagram of the system ABC. In contrast with binary systems, the equilibrium compositions of the solid and liquid phases in a ternary system are not determined uniquely. After fixing the composition of A on the solid side of the interface, the three other equilibrium compositions are found by equalizing the chemical potentials and the Grand potential of the two phases. In particular, we obtain the composition of B in the liquid c_B^l shown in black in Supplementary Fig. 1.b

As depicted in Supplementary Fig. 1.b, our simulations show that the concentration c_B^l decreases with c_A^p as predicted by the equilibrium phase diagram. However, the relation extracted from our simulations underestimate significantly the value of c_B^l compared to the bulk phase diagram. This is due to the gradient terms of the free energy that strongly influence the interfacial concentration profiles, modifying the equilibrium condition. It has been checked that, for weaker gradient terms, the simulated data points reach the phase diagram prediction.

On Supplementary Fig. 1.b is also plotted a data point extracted from the 2D dealloying simulation performed with $c_0 = 15\%$ (depicted in Fig. 3.a of the main article) where c_A^p is taken as the maximum value of the interfacial ridge of A and c_B^l is measured at the bottom of the liquid fingers. The slight discrepancy between this data point and the results extracted from our 1D simulations is attributed to the influence of the interface curvature on the equilibrium compositions through the Gibbs-Thomson effect.

The relation between c_B^l and c_A^p obtained from our 1D simulations and plotted in Supplementary Fig. 1.b can be approximated with a decreasing exponential of the form:

$$c_B^l(c_A^p) = c_{B,0}^l \exp\left(-c_A^p/c^*\right) \tag{1}$$

The velocity of the interface is then given by the flux of B leaving the interface and can be approximated by a linear function of c_B^l. Thus, we recover the exponential relation between the interface velocity v_i and the concentration c_A^p:

$$v_i(c_A^p) = v_0 \exp\left(-c_A^p/c^*\right), \tag{2}$$

described in Fig. 2 of the main article.

Supplementary Note 2: Phase-field model

We use a phase-field model for ternary system A-B-C. This type of model rely on the coupling between concentration fields of the different species ($c_A(r)$, $c_B(r)$ and $c_C(r)$ with $c_C(r) = 1 - c_A(r) - c_B(r)$) and an order parameter $\phi(r)$ describing the crystalline order of the phase: $\phi(r) = 0$ (respectively $\phi(r) = 1$) if r is in the liquid (solid). The free energy functional of the system is written as:

$$\mathcal{F} = \int_V dV f = \int_V dV \Big\{ f_\phi + f_c(\phi, \{c_i\}) + f_g \Big\}. \tag{3}$$

The free energy density f_ϕ is expressed as the sum of a gradient term $\frac{\sigma_\phi}{2}|\nabla\phi|^2$, penalizing the spacial variations of the field ϕ, and a double-obstacle potential $f_{do}(\phi)$ defined as:

$$f_{do}(\phi) = \begin{cases} +\infty & \text{for } \phi < 0 \\ \lambda_\phi \phi(1-\phi) & \text{for } 0 \le \phi \le 1 \\ +\infty & \text{for } \phi > 1 \end{cases} \tag{4}$$

The values of σ_ϕ and λ_ϕ (see Supplementary Table 1) are chosen to obtain a solid/liquid interface of width $w_i = 2$ nm and interfacial energy $\gamma = 0.2$ J m^{-2}.

The term $f_c(\phi, c_i)$ accounts for the thermodynamic properties of a mixture between components A, B and C modeled like a regular solution in both liquid and solid phases:

$$f_c(\phi, \{c_i\}) = \sum_{i=A,B,C} \left(\phi c_i L_i \left(\frac{T - T_i}{T_i} \right) + \frac{k_B T}{V_a} c_i \log(c_i) \right) + \Omega c_A c_C \tag{5}$$

The first term introduces a coupling between the order parameter ϕ and the composition c_i via the latent heat of fusion of the pure elements L_i and the relative difference between the temperature of the system and the melting temperatures of the pure elements T_i. The values of these parameters listed in Supplementary Table 1 are chosen to model a mixture of Ta (A), Ti (B) and Cu (C). The second term is simply the entropic contribution of the free energy where k_B is the Boltzmann constant and V_a the atomic volume assumed to be independent of the phase (solid or liquid) and the composition. For simplicity reasons, we assume that the couples (A,B) and (B,C) can be modeled with ideal solution models, having a negligible enthalpy of mixing. On the other hand, the strong partitioning between A (Ta) and C (Cu) is modeled with a regular solution model and we denote Ω the enthalpy of mixing between these two species.

Finally, f_g denotes the free energy density associated with the spatial variations of the composition profiles and is expressed as a sum of gradient terms on the composition fields $f_g = \sum \frac{\sigma_i}{2}|\nabla c_i|^2$.

The phase diagrams of the binary systems (C,B), (B,A) and (A,C) can be computed from the free energy density f_c using the common tangent construction and are displayed in Supplementary Fig. 7. While the binary systems (C,B) and (B,A) have simple lens-shape phase diagrams, the system (A,C) is monotectic and display phase separation in the liquid phase.

In our simulations, the value of the temperature is $T_{sim} = 1775$ K (horizontal dashed line in Supplementary Fig. 7) for which our simplified thermodynamic model reproduces approximatively the phase-diagram of the ternary system Ta-Ti-Cu at the temperature $T_{exp} = 1513$ K used in experiments. In particular, at $T_{sim} = 1775$ K, the equilibrium concentration of B in the liquid C for the binary phase diagram (C,B) is close to the equilibrium concentration of Ti in the liquid Cu read on the experimental phase-diagram at the temperature $T_{exp} = 1513$ K.

We now turn to the dynamic equations for the fields $\{c_i\}$ and ϕ. The concentrations fields c_A and c_B are assumed to follow Cahn-Hilliard equations:

$$\partial_t c_i = \nabla \cdot M_{ij} \nabla \mu_j, \tag{6}$$

where $\mu_j = \delta \mathcal{F}/\delta c_j$ is the chemical potential of element j and M_{ij} are the components mobility matrix expressed as $M_{ij} = M_0(\phi) c_i(\delta_{ij} - c_j)$ with $M_0(\phi) = (1 - \phi(x))M_l$ a linear function of ϕ chosen such that the mobility is frozen in the solid and reaches $M_l = V_a D_l / k_B T$ in the liquid. This expression of M_l insures that, in the dilute limit, Eq. (6) converges to a simple Fickian diffusion $\partial_t c_i = D\nabla^2 c_i$ for the different concentration fields.

The order parameter ϕ is assumed to follows a simple dissipative dynamics for which the variation of ϕ is directly proportional to the functional derivative $\frac{\delta \mathcal{F}}{\delta \phi}$:

$$\partial_t \phi = -L_\phi \frac{\delta \mathcal{F}}{\delta \phi}. \tag{7}$$

We assume that the solid/liquid interface is atomically rough such that the attachment / detachment kinetics of atoms at the interface is fast compared to their diffusion in the liquid. In other words, the evolution of the phase-field (Eq. (7)) happens on a much shorter time scale than the diffusion (Eq. (6)). To insure this separation of time-scales, the parameter L_ϕ is chosen to satisfy $L_\phi \gg M_l/w_i^2$. In practice, we take $L_\phi = 10M_l/w_i^2$ and it has been checked that a larger value of L_ϕ does not change the results.

We note here that the double-obstacle potential of Eq. (4) is preferred to a generic double-well potential because it insures that the thermodynamic and kinetic properties reach their values for the liquid and solid phases at a small distance on both sides of the interface. This is consistent with molecular dynamics simulations performed on Al-Pb solid/liquid interfaces [2].

The equations are normalized with the characteristic length w_i (the solid/liquid interface width) and the characteristic time w_i^2/D_l. Eqs. (6) and (7) are discretized in space on a finite difference grid with a regular spacing dx and in time with a forward Euler scheme of step dt. The calculations are performed using a parallel GPU code.

The 1D simulations used to produce Fig. 2.a, b and c of the main article are performed on a large domain, representative of an infinite bath of C liquid. To insure the convergence of the integration, we take $dx = 0.1$ and $dt = 10^{-5}$ (in dimensionless units). Because of the discrete nature of the numerical implementation, the interface velocity and the interfacial concentrations are not smooth functions of time but present a jagged behavior. Before plotting Figs 2.b and c, the velocity and interfacial concentrations are averaged on small time windows during which the interface moves an amount dx. The simulation displayed in Fig 2.d has been performed with the same fine discretization and starting with an alloy of uniform composition $c_0 = 10\%$ in contact with a large bath of C liquid. Initially, the field ϕ is slightly perturbed by adding a uniformly distributed white noise of amplitude ± 0.025. This small initial perturbation triggers the instability leading to the formation of high-C domains along the interface.

The 2D and 3D simulations displayed or used in Fig. 1, 3, 4 and 5 of the main article are performed with a coarser discretization $dx = 0.25$ in order to reach a relevant domain size while keeping a reasonable computational time. In those simulations, the initial composition of the alloy is perturbed around its average value by adding a uniformly distributed white noise of amplitude ± 0.025. This noise is introduced to mimic composition inhomogeneities in the solid. 2D simulations are performed on a finite difference grid of dimensions $N_x = 768$ and $N_y = 512$, simulating a domain size 256x384 nm^2. 3D simulations are performed with dimensions $N_x = 256$ and $N_y = N_z = 192$, simulating a domain size 128x96x96 nm^3. For both

2D and 3D simulations, the concentration at the liquid edge of the domain (i.e. at $x = N_x$) is maintained fixed close to pure A in order to mimic the mixing due to the electromagnetic stirring occurring in experiments. A “no flux” boundary condition is imposed at the solid edge of the domain ($x = 0$) and periodic boundary conditions are imposed in other directions.

Supplementary Note 3: Diffusion-coupled growth model

In this section, we detail the simple 1D diffusion model proposed to describe the coupled growth regime and the derivation of Eq. (1) of the main article.

The coupled growth regime is modeled by the simplified geometry depicted on Supplementary Fig. 2.b. We consider that the solid-liquid interface can be considered planar and we introduce the coordinate u running along the interface (see Supplementary Fig. 2.b). We also assume that the system reaches a pseudo-stationary regime with a constant dealloying rate v_i. During a time Δt, the solid/liquid interface advances a distance $v_i \Delta t$ and an amount $c_0 v_i \Delta t/\xi$ of A is accumulated at the solid/liquid interface on a length-scale ξ. This amount is confined within the interface and diffuses over a distance $\lambda_0/2$ to the A-rich solid ligaments during the same time Δt. Thus, the interfacial concentration of A noted $c_i(u)$ follows a stationary 1D diffusion equation with a source term:

$$D_i \frac{d^2 c_i}{du^2} + \frac{v_i c_0}{\xi} = 0, \tag{8}$$

where D_i is the interfacial diffusion constant accounting for the reduced diffusivity in the solid/liquid interface. Integrating Eq. (8) in u, we find that the interfacial concentration profile has a quadratic profile

$$c_i(u) = c_{eq} + \frac{v_i c_0}{2 D_i \xi} \left(\frac{\lambda_0^2}{4} - u^2 \right), \tag{9}$$

where c_{eq} is the concentration reached at the interface of the high composition domain (see Supplementary Fig. 2.b). Taking Eq. (9) at $u = 0$, we deduce a relation between the ligament spacing λ_0 and the dealloying rate v_i:

$$\lambda_0^2 = \frac{8 D_i \xi \Delta c}{c_0 v_i}, \tag{10}$$

where $\Delta c = c_t - c_{eq}$ is the difference between the extrema of the concentration profile (see Supplementary Fig. 2.b).

While the length ξ is rather straightforward to estimate from the interfacial concentration profile, the values of the parameters D_i and Δc can be more troublesome to evaluate because the diffusivity varies within the solid/liquid interface and the interfacial concentration profile has a complex shape. A close observation of the profiles shows that the concentration fluxes of A are maximum along the iso-line $\phi = 0.5$ rather than at the ridge of the concentration profile where the diffusivity is smaller. The value of D_i is thus taken for $\phi = 0.5$ ($D_i = 0.5\, D_l$) and the difference Δc is estimated from the concentration profile of A taken along the the iso-line $\phi = 0.5$. This concentration profile is depicted in Fig. 3.c of the main article.

Supplementary Note 4: Activation energy and rate limiting mechanism

To investigate the influence of the temperature on the dealloying kinetics, dealloying experiments have been performed at four different temperatures ($T = 1433$ K, $T = 1513$ K, $T = 1578$ K, $T = 1633$ K) for different values of c_0 (30%, 45%). For each sample, the dealloying depth x_i is measured from SEM observations. The results are then fitted against the following relation:

$$x_i = \left(Ae^{-E_a/kT}\right)^n \tag{11}$$

where the values of A, E_a and the exponent n are determined through the fitting procedure. Supplementary Fig. 3 shows the dealloying depth function of the rescaled time $te^{-Ea/kT}$, showing the good quality of the fit.

The values obtained for the exponent n are found to be close to 1/2 ($n = 0.38$ for $c_0 = 30\%$ and $n = 0.41$ for $c_0 = 45\%$), suggesting that the dealloying depth is controlled by a diffusion mechanism. The fact that we find values smaller than 1/2 can be attributed to the presence of the dealloyed structure and the evacuation of Ti from the dealloyed ligaments, leading to a slowing down of the kinetics.

More importantly, the activation energy E_a obtained from the fits is found to be $E_a = 0.69$ eV for both composition. This value is very close to $E_{Ti \to Cu} = 0.715$ eV, the activation energy of Ti diffusion in Cu liquid, measured experimentally in previous studies [1], showing that the limiting process is most certainly the diffusion of Ti in liquid Cu.

We note here that for higher initial Ta composition of the alloy (e.g. $c_0 = 60\%$), the activation energy is found to be much higher (above 1 eV), suggesting that another mechanism might become rate-limiting.

Supplementary Note 5: Linear profile approximation

In the dealloying experiments, the heating of the liquid metal is performed through electromagnetic induction. A consequence is the mixing of the liquid outside the dealloyed structure due to strong convection currents. However, inside the dealloyed region of the sample, the convection currents are stopped by the fine structure. Thus, we consider that the liquid concentration is fixed close to pure Cu outside of the dealloyed structure while it follows a diffusive kinetics inside the dealloyed region.

This is supported by the concentration profiles measured experimentally. After partial dealloying of a sample of composition $c_0 = 30\%$ and solidification of the liquid metal, energy-dispersive X-ray spectroscopy is performed to determine the composition profiles of the different phases. Supplementary Fig. 4 shows the concentration profiles of Ti in the Cu-Ti phase function of the coordinate normal to the dealloying front for three different dealloying times. Even though the experimental measurements are quite scattered, the composition can be considered to be maintained close to pure Cu in the vicinity of the edge of the dealloyed structure.

To mimic the situation of a fixed composition outside of the dealloyed structure, simulations are performed with a fixed concentration close to pure A at the liquid edge of the domain. Supplementary Fig. 5 shows the average concentration of B (Ti) in the B-C liquid phase obtained numerically for 2D dealloying simulations performed with $c_0 = 15\%$ and $c_0 = 30\%$.

Supplementary Figs. 4 and 5 show that the concentration of the dealloyed element Ti (B) at the dealloying front noted c_B^l does not change significantly during the dealloying process: it is maintained at $c_B^l = 50$ % in experiments and $c_B^l = 18$ % in the simulations. The significative

difference between these values is attributed to our simplified thermodynamical model and the strong gradient terms used in our simulations that have a significant influence on the interface equilibrium (see Supplementary Note 1).

Thus, diffusion of B in C in the dealloyed structure can be considered to be subjected to boundary conditions at the dealloying front ($c_B = c_B^l$) and at the edge of the dealloyed structure ($c_B = 0$). It is thus natural to assume that the diffusion profile of Ti(B) can be considered to be linear between these two boundaries:

$$c_B(x, x_i) = c_B^l \left(1 - \frac{x}{x_i}\right), \tag{12}$$

where the origin of the x axis is now taken at the dealloying front and x_i is the dealloying depth, i.e. the distance between the dealloying front and the edge of the dealloyed structure. The profiles obtained both experimentally (Supplementary Fig. 4) and numerically (Supplementary Fig. 5) show that this linear profile approximation is reasonable.

Supplementary Note 6: Dealloying rate and diffusion limited kinetics

Because the dealloying process is limited by the diffusion of Ti (B) in Cu (A) liquid, the dealloying rate v_i is simply given by the flux of Ti (B) leaving the solid/liquid interface, evacuating the excess of Ti (B) between the solid composition $1 - c_0$ and the liquid composition c_B^l at the interface. For simplicity reasons, we neglect the role of the Ta-rich ligaments as obstacles to the diffusion in the liquid. The dealloying rate is given by:

$$v_i = \frac{dx_i}{dt} = \frac{1}{1 - c_0 - c_B^l} J_B \tag{13}$$

where J_B is the flux of the dealloyed element B leaving the dealloying front. It can simply be computed from the linear composition profile of Eq. (12):

$$J_B = -D_l \left.\frac{dc_B(x)}{dx}\right|_{x=0} = \frac{D_l c_B^l}{x_i} \tag{14}$$

Finally, Eq. (13) simply becomes

$$v_i = \frac{dx_i}{dt} = \frac{D\alpha}{2x_i}, \tag{15}$$

where $\alpha = 2c_B^l/(1 - c_0 - c_B^l)$ is constant for a given alloy composition. The integration of this simple differential equation in time yields the evolution of the dealloying position in time:

$$x_i(t) = \sqrt{\alpha D_l t}, \tag{16}$$

presenting a square-root behavior, characteristic of diffusion limited dynamics.

We note here that a similar square-root behavior can be derived by considering that the dealloyed element diffuses into an infinite bath of Cu (B). Indeed, the resolution of the diffusion equation in a semi-infinite domain yields that the Péclet number of the problem $p = x_i v_i / 2D$ depends only on the concentration c_B^l via a transcendental equation. The time integration for a fixed Péclet number yields a square root dynamics similar to Eq. (16).

Supplementary Note 7: Experimental methods

Ti-Ta alloys were prepared in-house by radio-frequency (RF) induction using an Ambrel Ekoheat 45 kW system by melting Ti (99.995 wt · %) and Ta (99.95 wt · %) pellets from Kurt Lesker in a water-cooled copper crucible from Arcast Inc. under flowing Ar (99.999 wt · %). Master ingots of ~ 30 g were made of the following compositions: Ta_5Ti_{95}, $Ta_{15}Ti_{85}$, $Ta_{25}Ti_{75}$, $Ta_{30}Ti_{70}$, $Ta_{35}Ti_{65}$, $Ta_{45}Ti_{55}$ and $Ta_{60}Ti_{40}$. Samples dedicated to liquid metal dealloying (roughly 6x2x10 mm^3) were cut from the master ingots using wire electrical discharge machining.

Two different dealloying experiments were performed in this study: immersion experiments (depicted in Supplementary Fig. 6.a), and static experiments (Supplementary Fig. 6.b). In both cases, the heating of the molten Cu is performed with a RF electromagnetic induction system.

For the immersion experiments, a 40 g molten Cu (99.99 wt · %) bath is prepared in a high-purity alumina crucible (cast in-house with materials from Cotronics) prior to immersion at a known temperature ($T = 1513$ K unless otherwise specified). The temperature is controlled with a Ircon Modline 5 infrared camera properly calibrated. Ta wires are spot-welded onto one end of the sample to allow the sample handling. Prior to immersion, the TiTa sample is brought inside the RF coils without touching the liquid bath to limit thermal gradients between the sample and the bath. The sample is immersed into molten Cu for a fixed time (ranging between 1 sec and 120 sec) before being removed. The removal of the sample from the liquid bath leads to the interruption of the dealloying process and allows the cooling down of the sample. This technique is applicable for samples with Ta compositions above 30 %, but structures obtained from alloys with lower Ta compositions had poor mechanical integrity and often fell apart when removing the sample from the bath. We attributed this to whether or not the dealloyed regions form connected networks of Ta ligaments (illustrated in Fig. 1 of the main article), a feature determined solely by the initial alloy composition. For this reason the immersion experiments are performed on $Ta_{30}Ti_{70}$, $Ta_{35}Ti_{65}$, $Ta_{45}Ti_{55}$ and $Ta_{60}Ti_{40}$ alloys.

To investigate lower Ta composition alloys, we use a slightly different experimental setup described in Supplementary Fig. 6.b. TiTa samples are placed in a high-purity alumina crucible along with 20 g of Cu (99.99 wt · %). The Cu is heated up until molten and rolls on to the TiTa ingot. After a fixed time, the RF power is turned off and the sample is allowed to cool down. We note that, under these conditions, we do not have precise control over the dealloying time. Experimental observations displayed in Fig. 1 of the main article are performed on samples delloyed with this experimental technique.

In both types of experiments, we obtain a finely structured composite of a Ta-rich ligaments network and a Cu-Ti phase.

These composite samples are mounted in Struers PolyFast conductive epoxy, and undergo: grinding steps of 400 grit, 600 grit; diamond polishing steps of 9 μm, 3 μm, 1 μm; and a final polishing step of 0.05 μm. Samples are then characterized using a JEOL scanning electron microscope (SEM) to observe the morphology, measure the dealloying depth and the average ligament size. The dealloying depth (Fig. 5.a of the main article) is determined by averaging about 40 measurements taken from four images from different regions of the sample. The average ligament spacing (Fig. 5.b of the main article) and ligament size (Fig. 5.c of the main article) are determined by averaging over 20 measurements.

The concentration profiles of the Cu-Ti phase presented in Supplementary Fig. 4 and discussed in Supplementary Note 5 are obtained by energy-dispersive X-ray spectroscopy.

Supplementary References

[1] D.B. Butrymowicz, J.R. Manning, and M.E. Read. Diffusion in Copper and Copper Alloys. Part III. Diffusion in Systems Involving Elements of the Groups IA, IIA, IIIB, IVB, VB, VIB, and VIIB. *J. Phys. Chem. Ref. Data*, 4(1):177–250, 1975.

[2] Y. Yang, D.L. Olmsted, M. Asta, and B.B. Laird. Atomistic characterization of the chemically heterogeneous al–pb solid–liquid interface. *Acta Mater.*, 60(12):4960–4971, 2012.
