# Supplementary figures and images for: Topology-generating interfacial pattern formation during liquid metal dealloying
Source: Nat Commun. 2015 Nov 19;6:8887. doi: 10.1038/ncomms9887 (PMC4673498; doi:10.1038/ncomms9887)

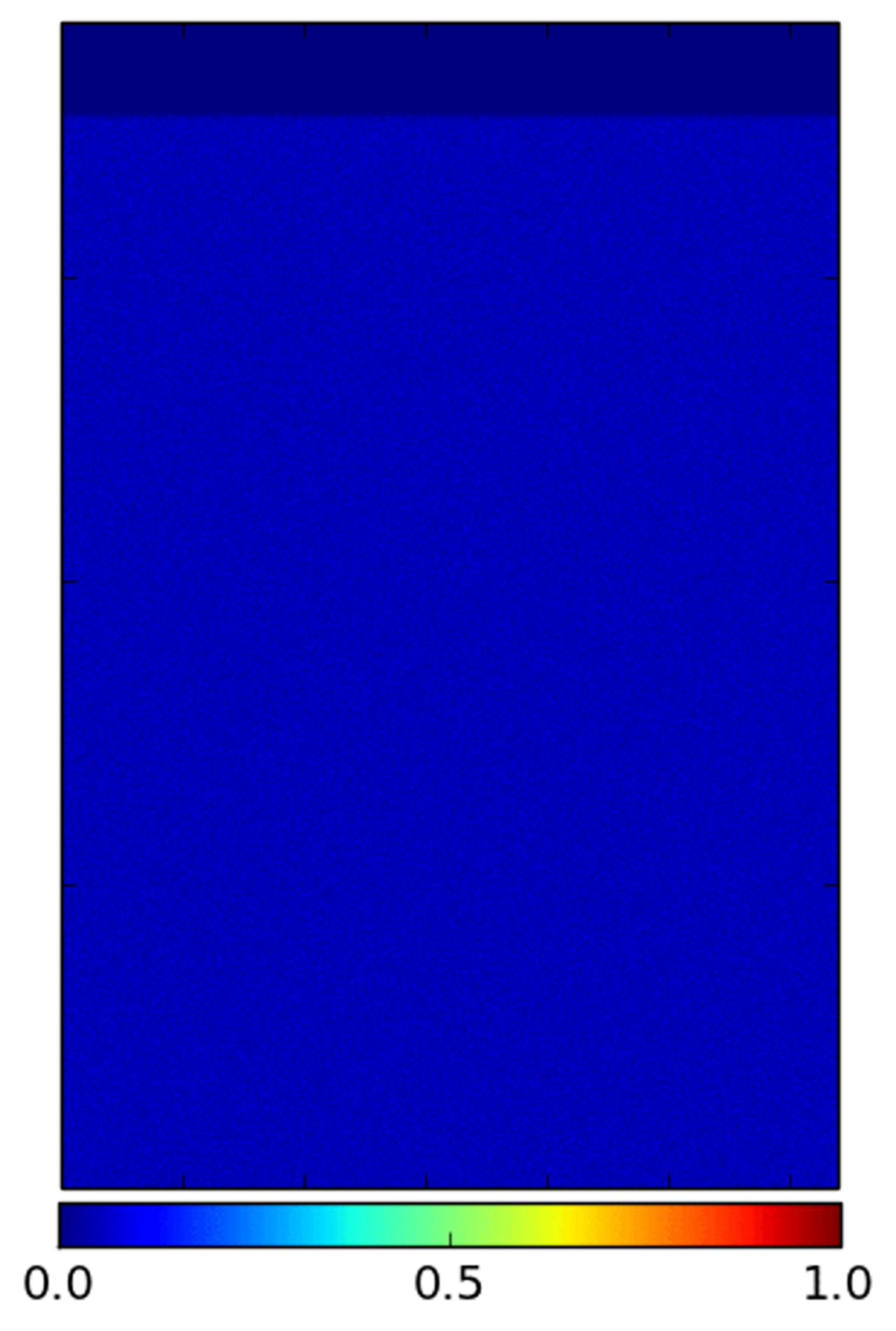

Supplement: Supplementary Movie 1 — This movie presents a 2D simulation (256 × 384 nm2) of the dealloying of a AB alloy with composition c0 = 5% in A in contact with pure C liquid, leading to the formation of non-connected islands. [file ncomms9887-s2.tif]

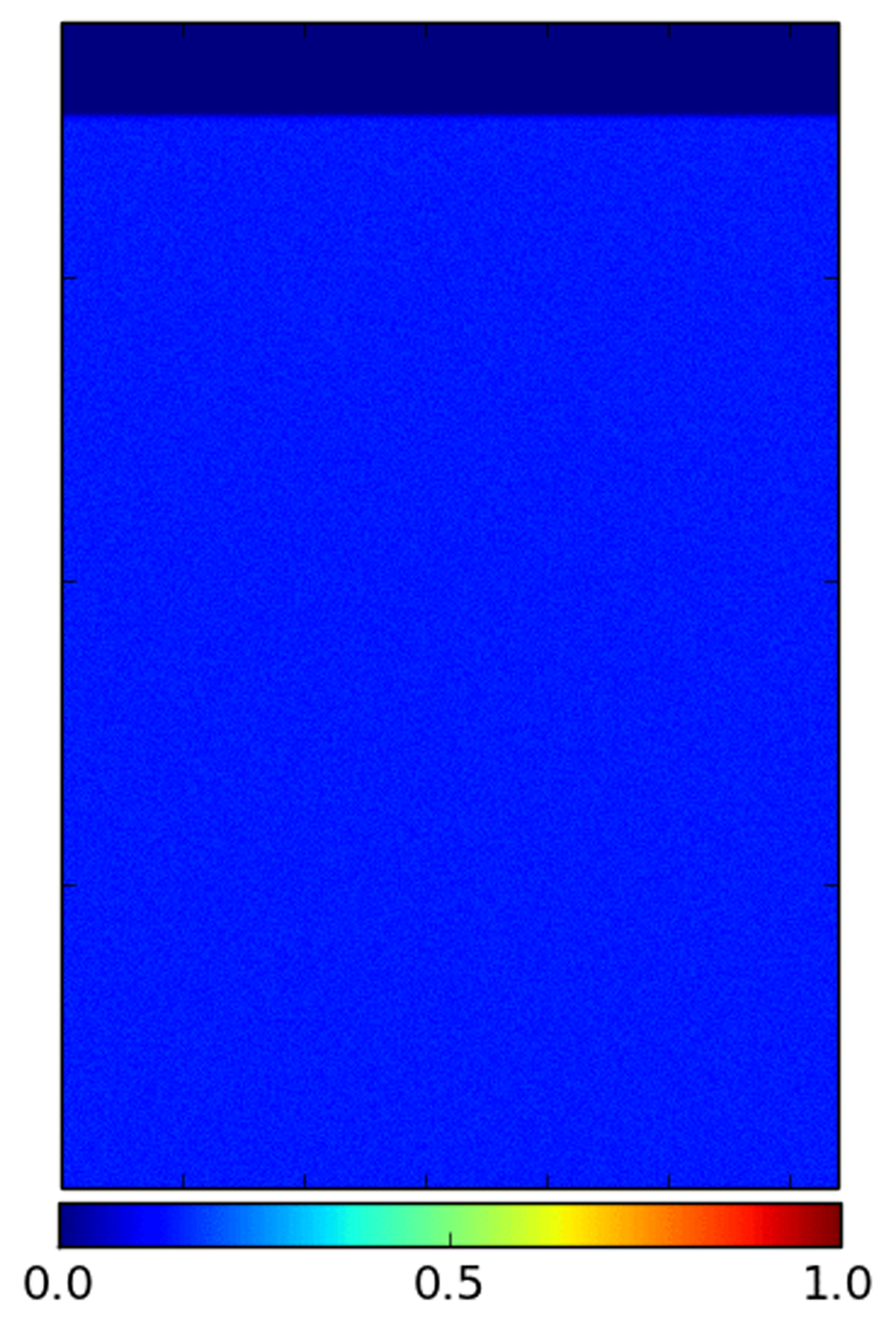

Supplement: Supplementary Movie 2 — This movie presents a 2D simulation (256 × 384 nm2) of the dealloying of a AB alloy with composition c0 = 15% in A in contact with pure C liquid, leading to the formation of elongated morphologies through the diffusion-coupled growth mechanism. [file ncomms9887-s3.tif]

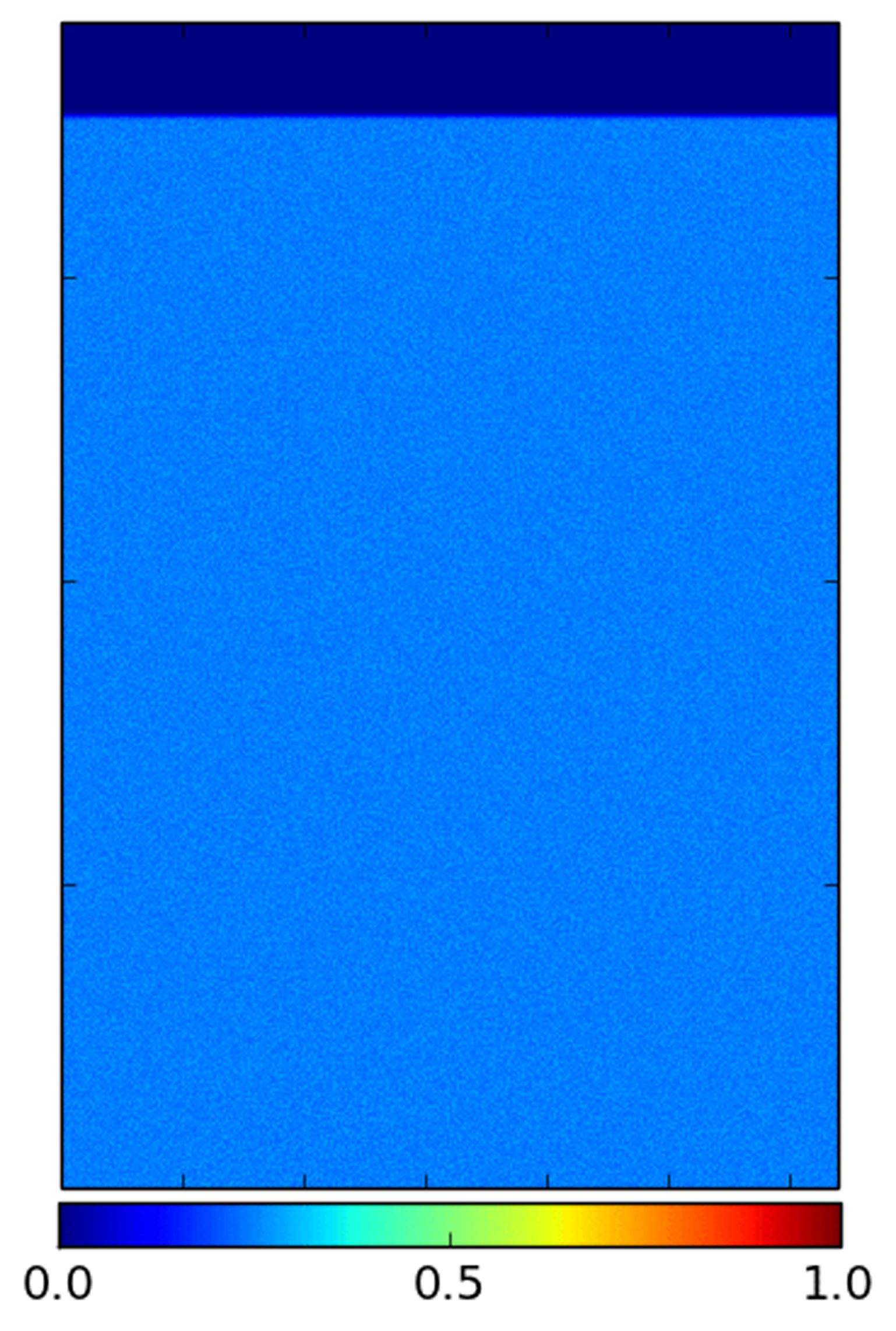

Supplement: Supplementary Movie 3 — This movie presents a 2D simulation (256 × 384 nm2) of the dealloying of a AB alloy with composition c0 = 25% in A in contact with pure C liquid, leading to the formation of a disordered structure. [file ncomms9887-s4.tif]

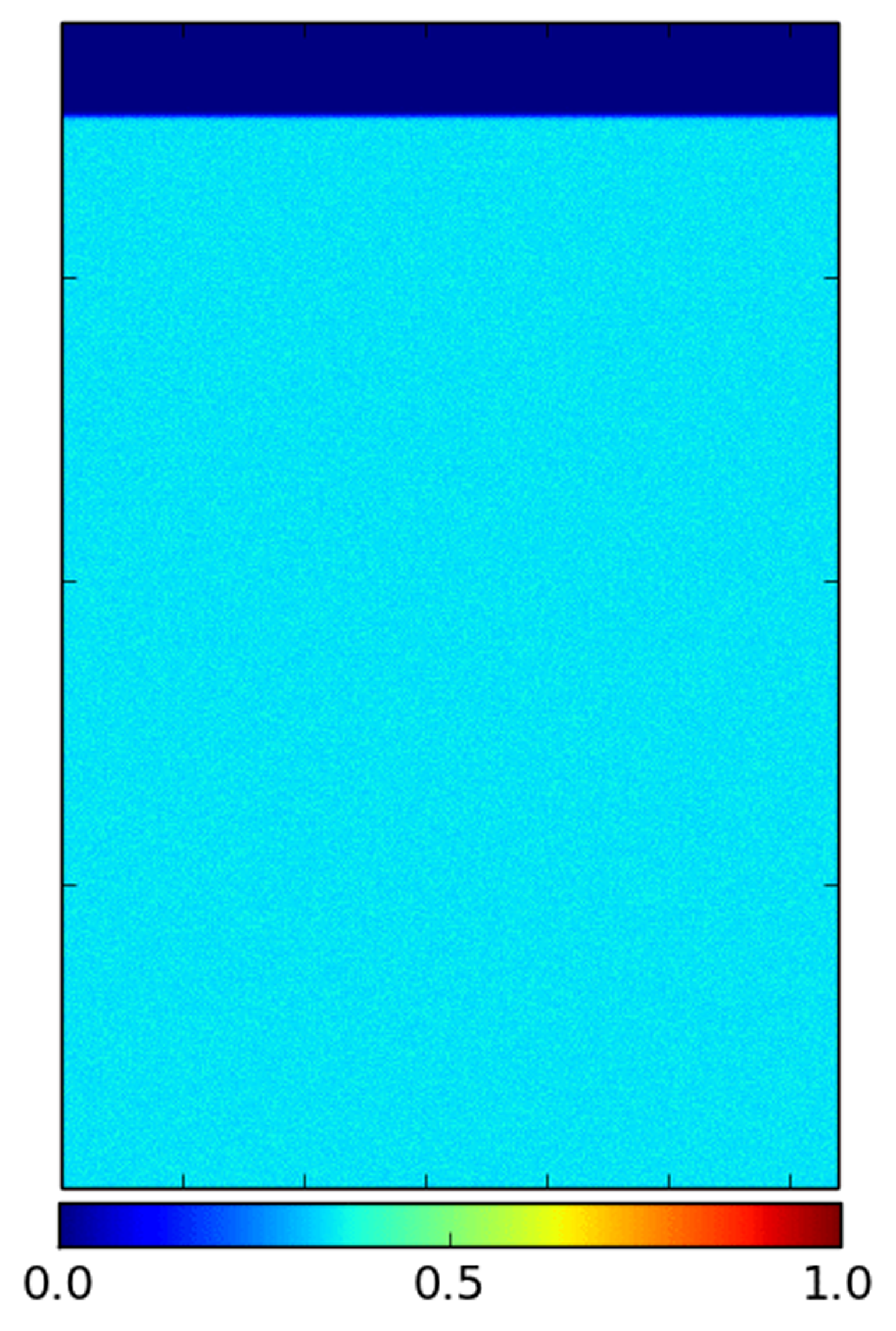

Supplement: Supplementary Movie 4 — This movie presents a 2D simulation (256 × 384 nm2) of the dealloying of a AB alloy with composition c0 = 35% in A in contact with pure C liquid, leading to the formation of a disordered structure. [file ncomms9887-s5.tif]

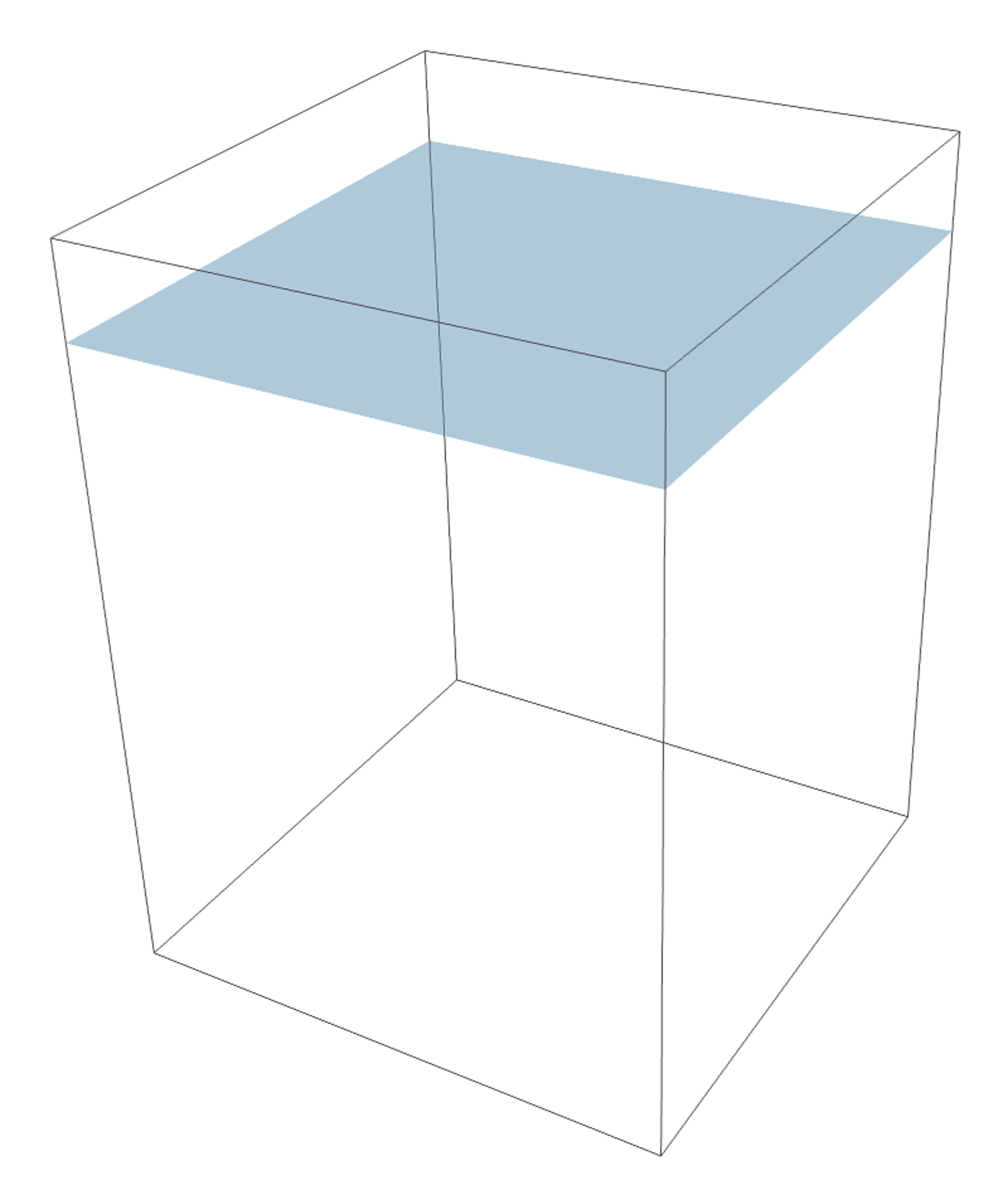

Supplement: Supplementary Movie 5 — This movie presents a 3D simulation (96 × 96 × 128 nm3) of the dealloying of a AB alloy with composition c0 = 5% in A in contact with pure C liquid, leading to the formation of non-connected blobs. [file ncomms9887-s6.tif]

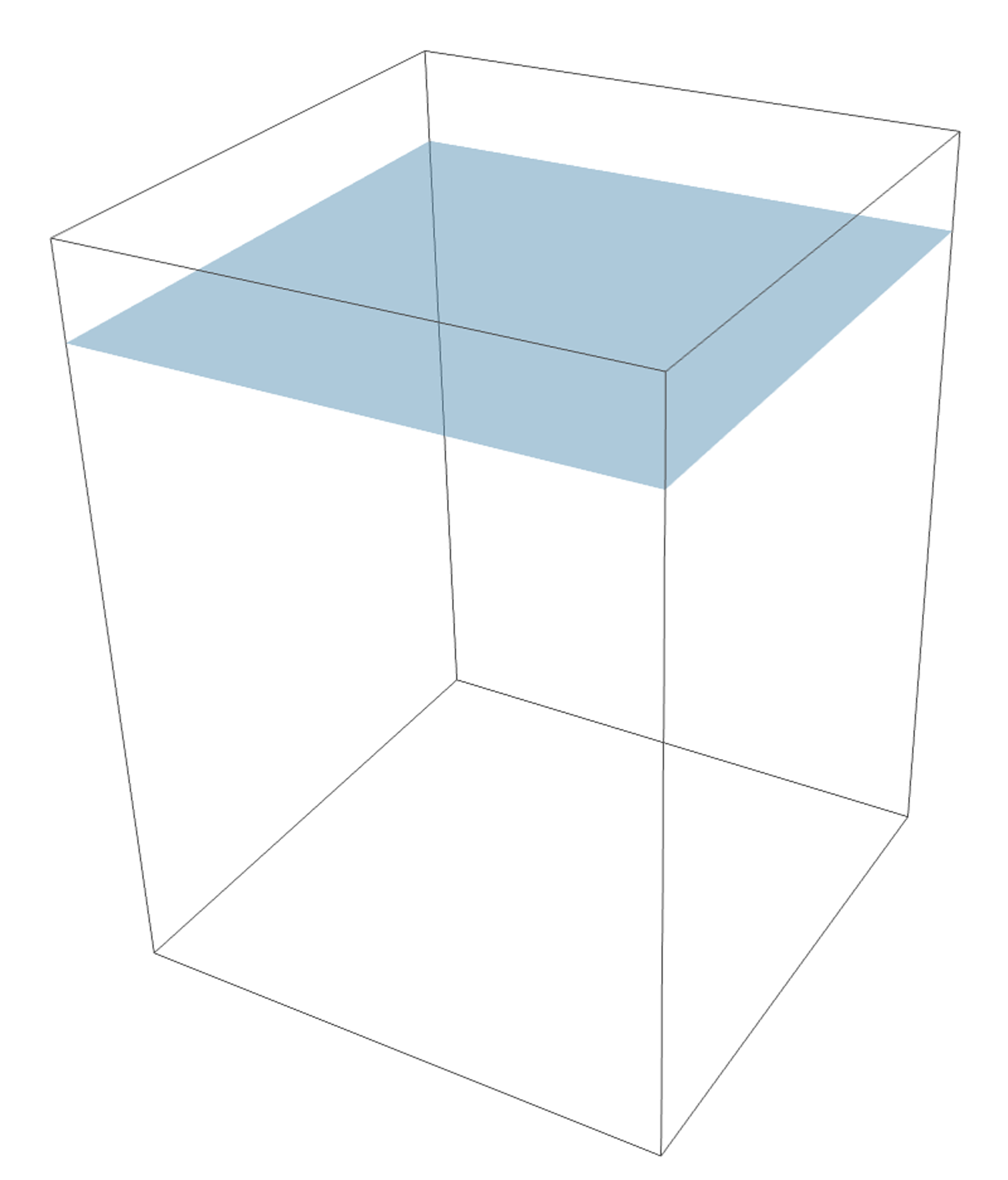

Supplement: Supplementary Movie 6 — This movie presents a 3D simulation (96 × 96 × 128 nm3) of the dealloying of a AB alloy with composition c0 = 15% in A in contact with pure C liquid, leading to the formation of elongated non-connected morphologies [file ncomms9887-s7.tif]

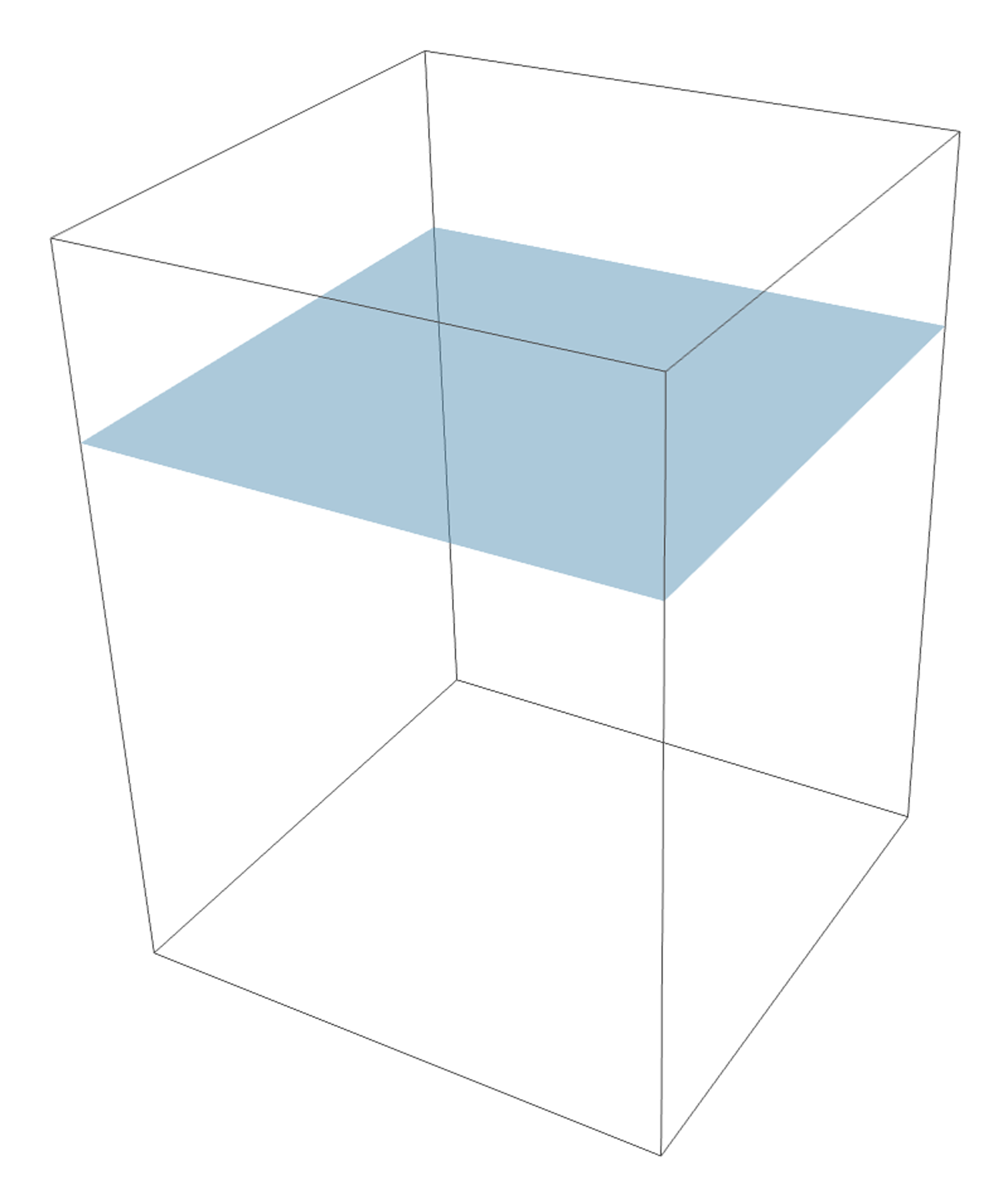

Supplement: Supplementary Movie 7 — This movie presents a 3D simulation (96 × 96 × 128 nm3) of the dealloying of a AB alloy with composition c0 = 25% in A in contact with pure C liquid, leading to the formation of a nanoporous connected structure. [file ncomms9887-s8.tif]

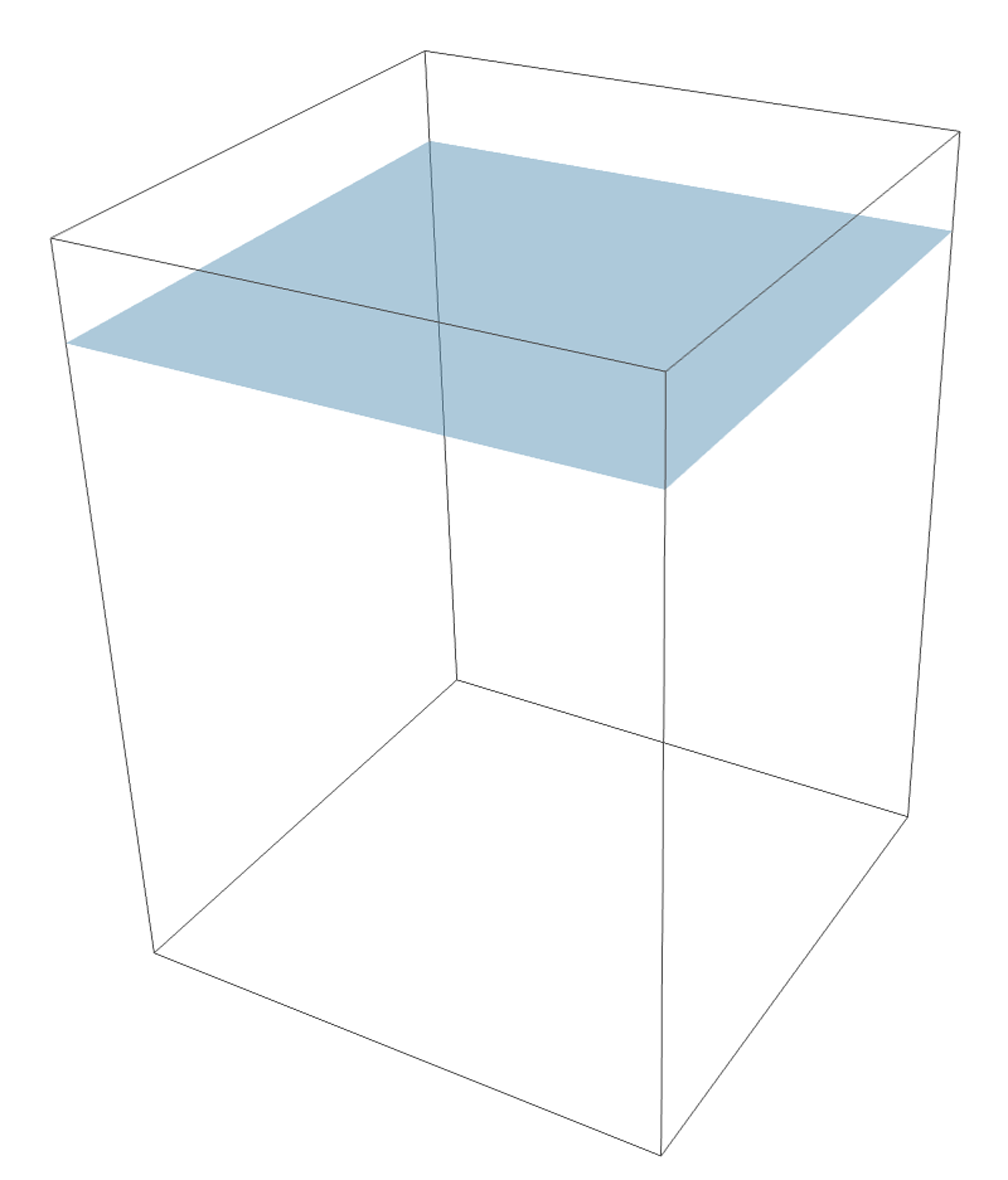

Supplement: Supplementary Movie 8 — This movie presents a 3D simulation (96 × 96 × 128 nm3) of the dealloying of a AB alloy with composition c0 = 35% in A in contact with pure C liquid, leading to the formation of a nanoporous connected structure. [file ncomms9887-s9.tif]
